# Heart non-specific effector CD4^+^ T cells protect from postinflammatory fibrosis and cardiac dysfunction in experimental autoimmune myocarditis

**DOI:** 10.1007/s00395-019-0766-6

**Published:** 2019-12-20

**Authors:** Martina Zarak-Crnkovic, Gabriela Kania, Agnieszka Jaźwa-Kusior, Marcin Czepiel, Winandus J. Wijnen, Jarosław Czyż, Björn Müller-Edenborn, Daria Vdovenko, Diana Lindner, Cristina Gil-Cruz, Marta Bachmann, Dirk Westermann, Burkhard Ludewig, Oliver Distler, Thomas F. Lüscher, Karin Klingel, Urs Eriksson, Przemysław Błyszczuk

**Affiliations:** 1 Cardioimmunology, Center for Molecular Cardiology, University of Zurich, Zurich, Switzerland; 2 Department of Rheumatology, Center of Experimental Rheumatology, University Hospital Zurich, Zurich, Switzerland; 3 Department of Medical Biotechnology, Jagiellonian University, Cracow, Poland; 4 Department of Clinical Immunology, Jagiellonian University Medical College, University Children’s Hospital, Wielicka 265, 30-663 Cracow, Poland; 5 Department of Cell Biology, Jagiellonian University, Cracow, Poland; 6 Department of Medicine, GZO-Zurich Regional Health Center, Wetzikon, Switzerland; 7 Clinic for General and Interventional Cardiology, University Heart Center Hamburg, Hamburg, Germany; 8 Institute of Immunobiology, Kantonsspital St. Gallen, St. Gallen, Switzerland; 9 Department of Cardiology, University Heart Center, University Hospital Zurich, Zurich, Switzerland; 10 Cardiopathology, Institute for Pathology and Neuropathology, University of Tubingen, Tubingen, Germany

**Keywords:** Heart, Myocarditis, Effector CD4^+^ T cells, Cardiac fibrosis, Th17 lymphocytes, Dilated cardiomyopathy, Experimental autoimmune myocarditis

## Abstract

Heart-specific CD4^+^ T cells have been implicated in development and progression of myocarditis in mice and in humans. Here, using mouse models of experimental autoimmune myocarditis (EAM) we investigated the role of heart non-specific CD4^+^ T cells in the progression of the disease. Heart non-specific CD4^+^ T cells were obtained from DO11.10 mice expressing transgenic T cell receptor recognizing chicken ovalbumin. We found that heart infiltrating CD4^+^ T cells expressed exclusively effector (*T*_eff_) phenotype in the EAM model and in hearts of patients with lymphocytic myocarditis. Adoptive transfer experiments showed that while heart-specific *T*_eff_ infiltrated the heart shortly after injection, heart non-specific *T*_eff_ effectively accumulated during myocarditis and became the major heart-infiltrating CD4^+^ T cell subset at later stage. Restimulation of co-cultured heart-specific and heart non-specific CD4^+^ T cells with alpha-myosin heavy chain antigen showed mainly Th1/Th17 response for heart-specific *T*_eff_ and up-regulation of a distinct set of extracellular signalling molecules in heart non-specific *T*_eff_. Adoptive transfer of heart non-specific *T*_eff_ in mice with myocarditis did not affect inflammation severity at the peak of disease, but protected the heart from adverse post-inflammatory fibrotic remodelling and cardiac dysfunction at later stages of disease. Furthermore, mouse and human *T*_eff_ stimulated in vitro with common gamma cytokines suppressed expression of profibrotic genes, reduced amount of α-smooth muscle actin filaments and decreased contraction of cardiac fibroblasts. In this study, we provided a proof-of-concept that heart non-specific *T*_eff_ cells could effectively contribute to myocarditis and protect the heart from the dilated cardiomyopathy outcome.

**Electronic supplementary material:**

The online version of this article (10.1007/s00395-019-0766-6) contains supplementary material, which is available to authorized users.

## Introduction

Myocarditis most commonly results from cardiotropic infections or tissue damage, followed by the activation of heart-specific autoimmunity [3, 34]. Lymphocytic myocarditis, characterized by extensive infiltration of lymphocytes and monocytes with signs of cardiomyocyte necrosis, represents the most common type of the disease [9]. Clinical presentation of the disease ranges from acute or chronic to fulminant myocarditis [35]. In about one-third of biopsy-proven cases, myocarditis progresses to dilated cardiomyopathy (DCM), characterized by extensive fibrosis, ventricular dilation and heart failure [9, 14]. The mechanisms underlying variable clinical outcomes of myocarditis remain, however, poorly understood.

Mouse models of experimental autoimmune myocarditis (EAM) reflect key aspects of the human disease [34]. In the classical EAM model, immunization with alpha-myosin heavy chain (α-MyHC) peptide and complete Freund’s adjuvant (CFA) induces myocarditis in susceptible mice. On follow-up, inflammation in the myocardium resolves and some mice develop progressive cardiac fibrosis, ventricular dilation and heart failure [4, 5, 21]. In the α-MyHC/CFA model, autoreactive CD4^+^ T cells play a central role in disease induction. Mechanistically, circulating α-MyHC-reactive CD4^+^ T cells, which escape the negative selection in the thymus [27] set the biological basis for EAM development. Following α-MyHC/CFA immunization, antigen-presenting cells (APCs) activate α-MyHC-reactive CD4^+^ T cells. During the initial phase, naïve CD4^+^ T (*T*_n_) cells become activated with antigen in the lymphatic system, where they undergo phenotypic changes. *T*_n_ cells convert into the effector (*T*_eff_) phenotype, expand and acquire new migratory properties [16]. In the effector phase, α-MyHC-reactive T_eff_ migrate into cardiac tissue, where they re-encounter α-MyHC antigen. This antigen-dependent response in the heart triggers production of proinflammatory cytokines, which induce myocarditis. Accordingly, adoptive transfer of CD4^+^ T cells isolated from α-MyHC/CFA immunized mice can convey disease from the host to recipient [30, 36]

The adverse post-inflammatory remodeling, including an excessive fibrotic response, characterizes progression of myocarditis to DCM and end-stage heart failure [6, 19]. In fact, accumulation of cardiac fibroblasts and their transformation into myofibroblasts is the hallmark of cardiac fibrosis [24]. CD4^+^ T cells and particularly the Th17 subset have been implicated in transition from myocarditis to DCM phenotype [2, 29]. Insight from other models also points to autoreactive CD4^+^ T cells as key mediators of cardiac fibrosis [15, 26].

In contrast to well-defined antigen-dependent responses, our understanding of antigen-independent mechanisms of CD4^+^ T cells is limited. It has been demonstrated that such antigen-independent stimulation of T_eff_, called also a bystander activation, could effectively contribute to CD4^+^ T cell expansion and function in a specific inflammatory condition [10]. Innate stimuli [18, 32] and cytokines of the common gamma family (γc-cytokines IL-2, IL-7, IL-15 and IL-21) [33] have been recognized as mediators of the bystander activation of CD4^+^ T cells. So far, however, the role of antigen-independent mechanisms and heart non-specific CD4^+^ T cells in myocarditis remained elusive.

## Methods

### EAM induction and adoptive T cell transfer

EAM was induced in 6–8 weeks old BALB/c or CD45.1-*tg* mice using an established protocol (see Supplemental Methods). In respective experiments, EAM mice were injected intravenously with either DO11.10^+^ *T*_eff_, DO11.10^+^ *T*_n_ or TCR-M *T*_eff_ (3–5 × 10^6^ per mouse) on days 17 and 20. For adoptive transfer, 6–8 weeks old BALB/c mice were sub-lethally irradiated (5.5 Gy) using a Gammatron (Co-60) and intravenously injected with 3–5 × 10^6^ CD45.1^+^ *T*_eff_, TCR-M *T*_eff_ and/or DO11.10^+^ *T*_eff_ per mouse. Sublethal irradiation depletes host’s T cells and thereby enables efficient engraftment and propagation of adoptively transferred T cells. Mice were euthanised by exposure to > 70% carbon dioxide or anesthetic overdose. All animal experiments were approved by local authorities and performed in accordance with Swiss federal and Polish law and the Guide for the Care and Use of Laboratory Animals, published by the US National Institutes of Health (NIH Publication, 8th Edition, 2011).

### Generation of *T*_eff_

Erythrocyte-lysed splenocytes/iLN cells were isolated from α-MyHC/CFA immunized CD45.1-*tg* mice at day 17 (CD45.1^+^ *T*_eff_), untreated TCR-M-*tg* (TCR-M *T*_eff_) or untreated DO11.10-*tg* (DO11.10^+^ *T*_eff_) mice. *T*_eff_ were generated by ex vivo CD4^+^ T cell activation with the respective antigen in the presence of APCs for 72 h (see Supplemental Methods). Activated CD4^+^ T cells purified by MACSorting using mouse anti-CD4 magnetic beads (Miltenyi) were used as *T*_eff_ for adoptive transfer experiments. For in vitro use, *T*_eff_ isolated from spleens/LN and hearts were additionally purified by FACSorting for CD4^+^CD44^hi^CD62L_low_ cells. *T*_n_ were isolated from spleens of DO11.10-*tg* mice and purified using CD4 positive MACSorting and FACSorting for CD4^+^CD44_low_CD62L^hi^ cells using FACS Aria III.

Human CD4^+^ T cell subsets were sorted from peripheral blood mononuclear cells (PBMCs) isolated from peripheral blood buffy coats of healthy donors (Blutspende Zurich, Switzerland), obtained with informed consent in accordance with the Declaration of Helsinki. PBMCs were isolated using Lympholyte (Cederlane) density gradient separation according to manufacturer’s instructions. CD4^+^ T cells were enriched by MACSorting using human anti-CD4 magnetic beads (Miltenyi). CD4^+^ T cell subpopulations were further sorted using FACS Aria III (BD Bioscience) based on the respective surface marker phenotypes: CD45RA^+^CD45RO^−^CCR7^+^CD27^+^ (*T*_n_) and CD45RA^−^CD45RO^+^CCR7^−^CD27^−^ (*T*_eff_).

### CD4^+^ T cell proliferation

CD4^+^ T cells were isolated from mouse erythrocyte-lysed splenocytes/LNs or human PBMCs by MACS or sorted from hearts digested with Liberase (Roche). CD4^+^ T cell subpopulations were isolated using FACS Aria III. Cells were labeled with 2.5 μM CFSE or 5 μM CellTrace Violet Cell Proliferation Kit (both Life Technologies) and either injected into recipient mice or cultured in vitro in the respective conditions (see Supplemental Methods). Cell proliferation was analyzed with LSRII Fortessa analyzer (BD Bioscience) and FlowJo software (Tree Star) using the Proliferation platform.

### CD4^+^ T cell and cardiac fibroblast co-culture

CD4^+^ T cell subpopulations were isolated from mouse or human CD4^+^ T cell fractions with FACS Aria III cell sorter and cultured on confluent mouse or human cardiac fibroblasts, respectively (see Supplemental methods).

### Flow cytometry and cell sorting

Single cell suspensions were prepared either from digested mouse hearts, erythrocyte-lysed splenocytes/LNs, or mouse and human PBMCs (see Supplemental methods). Freshly isolated or cultured cells were subjected to CD4 positive MACSorting and/or stained with the appropriate combination of fluorochrome-conjugated mouse/human antibodies (see Supplemental methods). Cells were analyzed with LSRII Fortessa analyzer. In the respective experiments, T cell fractions were sorted with FACSAria III (purity > 98%). For intracellular protein staining, cardiac fibroblasts were stained with anti-mouse/human αSMA (Sigma) and anti-human collagen I-biotin (Acris) antibodies (see Supplemental methods) and analyzed with LSRII Fortessa analyzer.

### Histopathology, immunohistochemistry and immunocytochemistry

Conventional hematoxylin/eosin and Masson’s trichrome staining were used to assess cardiac inflammation and fibrosis, respectively (see Supplemental methods). Immunopositive cells (for CD3, CD45, CD45.1, CD90.1) and fibrotic markers (Masson’s Trichrome; αSMA; periostin, vimentin, both Abcam) were quantified using Olympus BX51 microscope and cellSens (Olympus) or Fiji software. Formalin fixed, paraffin embedded human heart tissue from 10 lymphocytic myocarditis patients were obtained from the Biobank of the Institute for Pathology and Neuropathology, University Hospital Tubingen in accordance with local ethical regulations and stained using rabbit anti-human CD4 (EPR6855, Abcam), mouse anti-human CD45RO (UCHL-1, eBioscience) and mouse anti-human CD45RA (HI100, eBioscience); see Supplemental Methods. Image acquisition was performed with a Zeiss AxioObserver Z1 widefield microscope and processed by ImageJ.

Human and mouse cardiac fibroblasts were stained with anti-mouse/human αSMA antibody and phalloidin (Sigma); see Supplemental methods. Image acquisition was performed with a Leica DM IRE2 microscope equipped with the Nomarski Interference Contrast and the Operetta high-content screening platform **(**Perkin–Elmer).

### Echocardiography

Transthoracic echocardiography was performed using a Vevo 2100 system equipped with 30-MHz transducer (VisualSonics). Anesthesia was induced by 5% isoflurane and confirmed by the absence of the withdrawal reflex of one of the hind paws. During echocardiogram acquisition isofluorane was reduced to 1.5–2%. The heart was imaged in the bidimensional (2-D) mode, in the parasternal long-axis, short-axis and apical 4-chamber views. Detailed description is available in the Supplemental methods.

### Statistics

Where relevant, data were analyzed by unpaired, two-tailed Student’s *t* test, one-way ANOVA followed by the Dunnett’s post hoc test (for normally distributed data), or by Mann–Whitney test (for nonparametric data). Differences were considered statistically significant for *p *< 0.05. All analyses were performed with GraphPad Prism 6 software and values are expressed as mean with SEM or SD.

For the extensive materials and methods we refer to the Supplemental methods.

## Results

### CD44^hi^CD62L_low_ cells represent the effector subset of CD4^+^ T cells in EAM

**Fig. 1:**
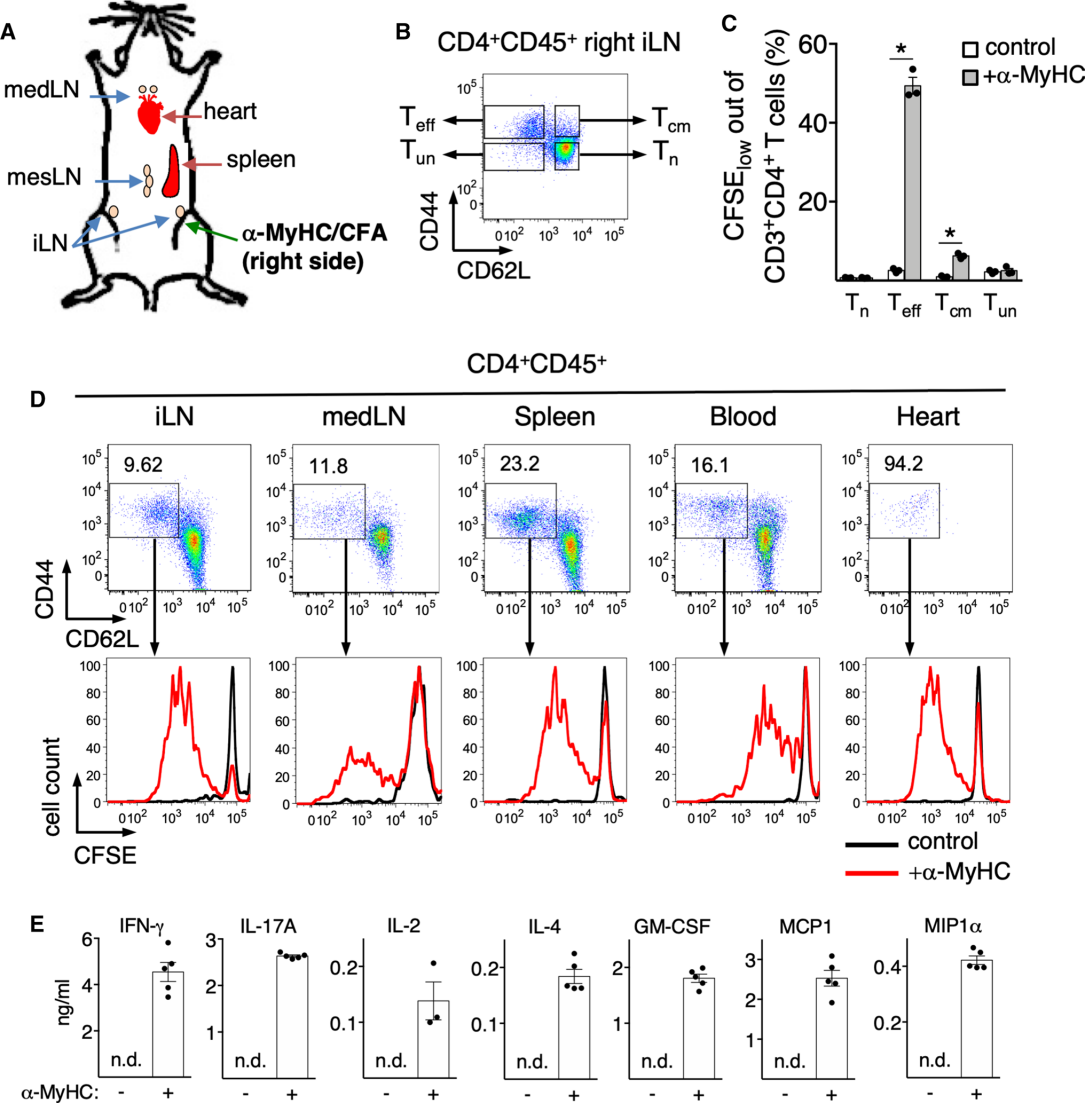
*T*_eff_ phenotype of heart-reactive CD4^+^ T cells in α-MyHC/CFA-induced EAM. BALB/c mice received subcutaneous injection of α-MyHC/CFA into the right inguinal region as shown in panel **a**. CD4^+^ T cells obtained from the right iLN 7 days after immunization were sorted into four fractions, labelled with CFSE and cultured for 3 days on APCs with or without α-MyHC. Panel **b** shows the gating strategy for CD4^+^ T cell subpopulations in viable CD4^+^ CD45^+^ population isolated from iLN: *T*_eff_ effector, *T*_cm_ central memory, *T*_n_ naïve and *T*_un_ undefined T cells. Percentage of proliferating CFSE-labelled CD4^+^ T cell subpopulations is quantified in panel **c**. Data are representative of three independent experiments, *p < 0.05 (unpaired Student’s *t* test). Panel **d** shows representative phenotype analyses of viable CD4^+^CD45^+^ cells and frequencies of CD4^+^CD44^hi^CD62L_low_ cells (*T*_eff_) in indicated organs at day 17 (active myocarditis). *T*_eff_ from respective organs were sorted, labelled with CFSE and stimulated with α-MyHC peptide in presence of APCs for 5 days. Proliferation is shown as dilution of CFSE dye in viable CD3^+^CD4^+^ population. Supernatant levels of indicated cytokines secreted by *T*_eff_ isolated from the heart are presented in panel **e**. Data are representative of two independent experiments. *iLN* inguinal lymph node, *mesLN* mesenteric lymph node, *medLN* mediastinal lymph node, *n.d*. not detected

To characterize the phenotype of inflammatory CD4^+^ T cells in human myocarditis, we analyzed cardiac biopsies of 10 patients with acute lymphocytic, virus-negative myocarditis (Supp. Table 1). Biopsies were evaluated using histology and immunofluorescence staining for detection of CD4, CD45RA (expressed on resting/naïve T cells) and CD45RO (expressed on activated and memory T cells). As shown by the representative staining (Supp. Fig. 1), nearly all CD4^+^ cells stained positive for CD45RO and negative for CD45RA, indicating that heart infiltrating CD4^+^ T cells in patients with acute lymphocytic myocarditis almost exclusively express a phenotype of activated *T*_eff_. Next, we characterized heart-specific CD4^+^ T cells in the mouse model of EAM. CD4^+^ T cells were isolated from blood and lymphoid organs of mice immunized with α-MyHC and CFA (Fig. 1a) 5–7 days after immunization (before myocarditis onset) and were further stimulated ex vivo for 3–5 days with α-MyHC antigen in the presence of APCs. Cells isolated from inguinal lymph nodes (LNs) located at the site of α-MyHC antigen injection showed the most pronounced α-MyHC-specific proliferation, suggesting local activation of heart-reactive CD4^+^ T cells (Supp. Fig. 2). To identify α-MyHC-responsive subsets, we sorted four populations of CD4^+^ T cells, based on their surface expression of CD44 and CD62L markers (Fig. 1b). A robust proliferative response to α-MyHC was observed only for the CD44^hi^CD62L_low_ *T*_eff_ population (Fig. 1c, Supp. Fig. 3). During the early phase of myocarditis (EAM day 17), α-MyHC-reactive *T*_eff_ were detectable not only in inguinal LNs, but also in mediastinal LNs, mesenteric LNs, blood, spleen and the heart (Fig. 1d). As expected, nearly all CD4^+^ T cells infiltrating cardiac tissue displayed the CD44^hi^CD62L_low_ *T*_eff_ phenotype. When isolated and exposed to α-MyHC antigen presented by APCs, these heart-infiltrating *T*_eff_ extensively proliferated and produced inflammatory cytokines (Fig. 1d, e).

### Heart non-specific *T*_eff_ effectively accumulate in the inflamed myocardium

**Fig. 2:**
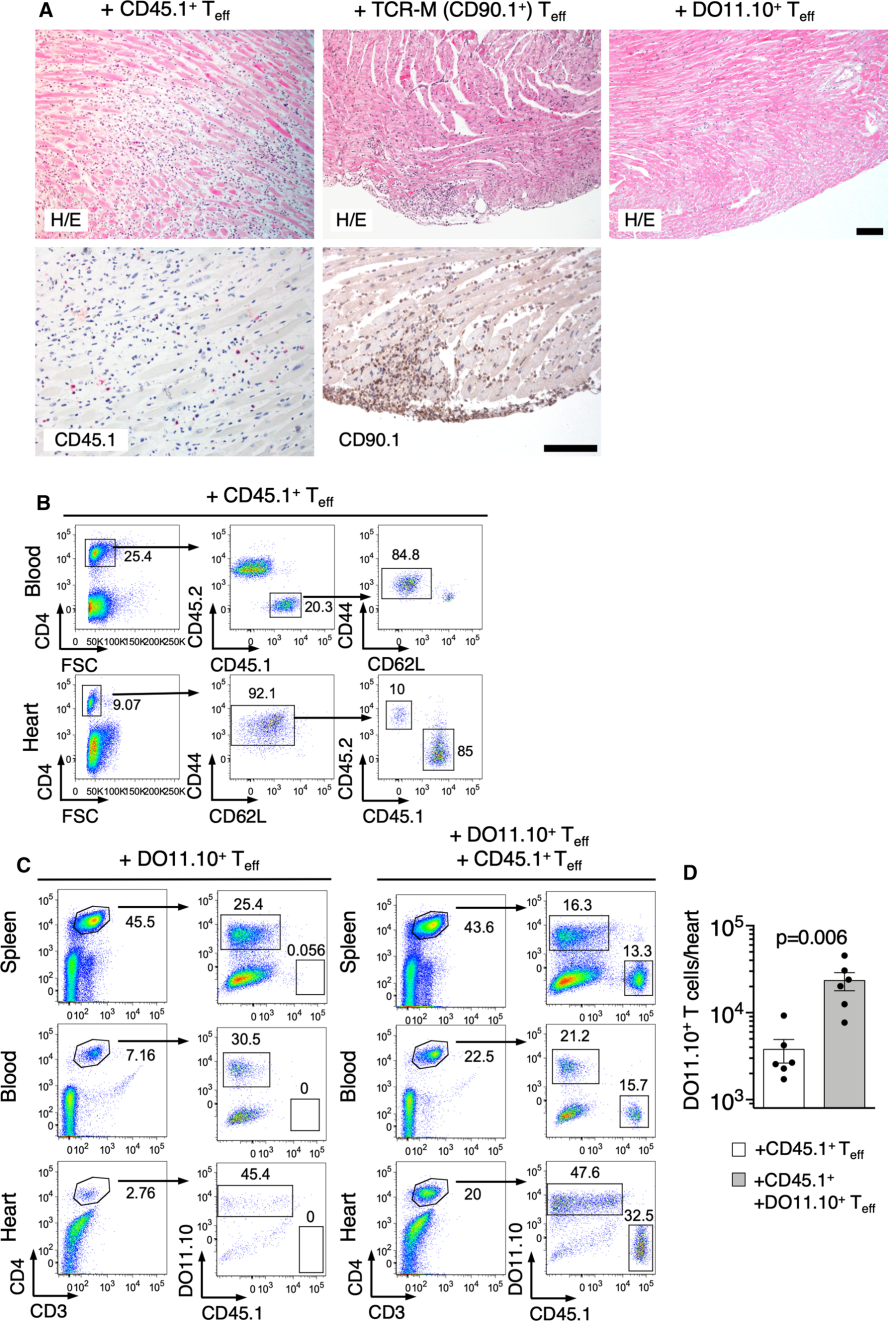
Cells with *T*_eff_ signature effectively infiltrate cardiac tissue. Indicated *T*_eff_ (3–5 × 10^6^) were adoptively transferred into sub-lethally irradiated BALB/c recipients. Top panel **a** shows representative heart histology of the respective recipient 10 days after adoptive transfer. Bottom panel **a** shows immunohistochemistry for CD45.1 and CD90.1 in hearts of indicated recipients. Scale bars = 100 μm. Panel **b** shows analysis of donor CD4^+^CD45.1^+^ cells in blood (top) and heart (bottom) 10 days after adoptive transfer of CD45.1^+^ *T*_eff_. Analysis of donor CD45.1^+^ and DO11.10^+^ *T*_eff_ in indicated organs 10 days after adoptive transfer is shown in panel **c** and quantification of total heart-inflammatory CD3^+^CD4^+^ DO11.10^+^ T cells is presented in panel **d**. Arrows show gating strategies and numbers indicate percentage of cells in the adjacent gate. Data are representative of six mice, *p* value calculated with unpaired Student’s *t*-test. CD45.1^+^ *T*_eff_—CD4^+^ cells from α-MyHC-stimulated (72 h) splenocytes/iLN cells of α-MyHC/CFA immunized CD45.1-*tg* mice (EAM d17); TCR-M *T*_eff_—CD4^+^ cells from α-MyHC-stimulated (72 h) splenocytes of TCR-M-*tg* (CD90.1^+^) mice; DO11.10^+^ *T*_eff_—CD4^+^ cells from OVA-stimulated (72 h) splenocytes of DO11.10-*tg* mice

Adoptive transfer of α-MyHC-re-stimulated CD4^+^ T cells isolated from inguinal LNs and spleens of α-MyHC/CFA immunized wild-type donor mice expressing CD45.1^+^ alloantigen, effectively induced myocarditis in sub-lethally irradiated CD45.2^+^ wild-type recipients (Fig. 2a). More than 80% of donor CD45.1^+^CD4^+^ cells displayed *T*_eff_ phenotype and these cells effectively accumulated in the hearts of the recipients (Fig. 2b), confirming the potential of heart-reactive *T*_eff_ to trigger myocarditis.

We adopted this T cell transfer model to study mechanisms of T cell trafficking to the heart. To address the differential role of heart-specific and heart non-specific T cells, we used CD4^+^ T cells from TCR-M and DO11.10 transgenic mouse strains, which expressed transgenic TCRs on their CD4^+^ T cells. In TCR-M mice, T cells react exclusively to α-MyHC antigen and these mice develop spontaneous myocarditis [31]. On the other hand, chicken ovalbumin (OVA)-reactive DO11.10^+^ CD4^+^ T cells from DO11.10 transgenic mice were used as a source of heart non-specific CD4^+^ T cells. To obtain *T*_eff_ for the adoptive transfer experiments, splenic and iLN cells from the respective donors were activated with their respective cognate antigen for 72 h and CD4^+^ T cells were sorted for experiments. Adoptive transfer of heart non-specific DO11.10^+^ *T*_eff_ failed to trigger inflammation (Fig. 2a), but DO11.10^+^ CD4^+^ T cells entered to a limited extent non-inflamed cardiac tissue (Fig. 2c). In contrast, heart-reactive TCR-M *T*_eff_ (which express CD90.1 alloantigen [31]) efficiently accumulated in the recipient hearts after adoptive transfer, although they induced myocarditis less efficiently than CD45.1^+^CD4^+^ T cells obtained from α-MyHC/CFA immunized mice (Fig. 2a).

Next, we co-transferred heart non-specific DO11.10^+^ *T*_eff_ together with α-MyHC-re-stimulated CD45.1^+^ *T*_eff_ from α-MyHC/CFA immunized mice. In inflamed hearts, we found not only myocarditis-inducing α-MyHC-reactive CD45.1^+^ *T*_eff_, but also a massive accumulation of heart non-specific DO11.10^+^ *T*_eff_ (Fig. 2c). The total amount of DO11.10^+^ *T*_eff_ was substantially higher in the inflamed hearts compared to the non-inflamed organs 10 days after adoptive transfer (Fig. 2d), demonstrating that the inflammatory response effectively attracted heart non-specific *T*_eff_ into the heart.

**Fig. 3:**
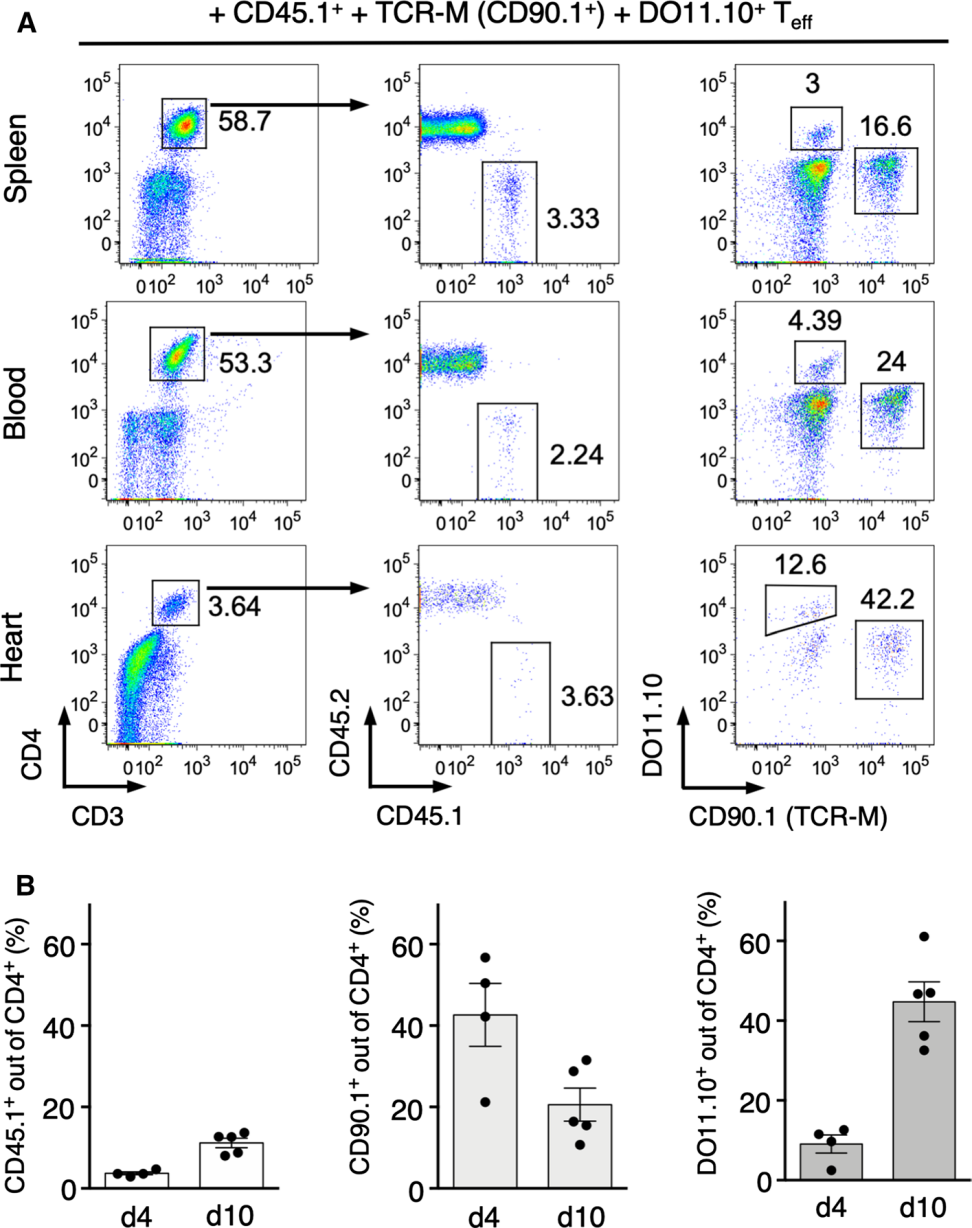
Differential kinetic of antigen-dependent and antigen-independent expansion of *T*_eff_ during myocarditis. CD45.1^+^ *T*_eff_ (4 × 10^6^), TCR-M *T*_eff_ (4.5 × 10^6^) and DO11.10^+^ *T*_eff_ (4.5 × 10^6^) cells were co-delivered into sub-lethally irradiated BALB/c recipients. Panel **a** shows analysis of donor CD4^+^ T cell frequency in indicated organs 4 days after adoptive transfer. Arrows show gating strategies and numbers indicate percentage of cells in the adjacent gate. Data are representative of four mice per time point. Panel **b** presents frequencies of indicated donor *T*_eff_ among heart-infiltrating CD4^+^ T cells at days 4 and 10 after adoptive transfer. Donor CD45.1^+^, TCR-M and DO11.10^+^ *T*_eff_ were generated as described in Fig. 2

To compare antigen-dependent and antigen-independent expansion of *T*_eff_ in the inflamed myocardium, we co-delivered α-MyHC-reactive CD45.1^+^ *T*_eff_ from α-MyHC/CFA immunized mice together with heart-specific TCR-M *T*_eff_ and heart non-specific DO11.10^+^ *T*_eff_. Four days after adoptive transfer, we detected all three injected *T*_eff_ in the recipient heart and TCR-M cells showed the most efficient heart-specific accumulation (Fig. 3a, b). However, at the peak of inflammation at day 10, DO11.10^+^ CD4^+^ T cells represented more than 50% of *T*_eff_ in inflamed hearts, becoming the dominant heart-infiltrating CD4^+^ T cell population (Fig. 3b).

**Fig. 4:**
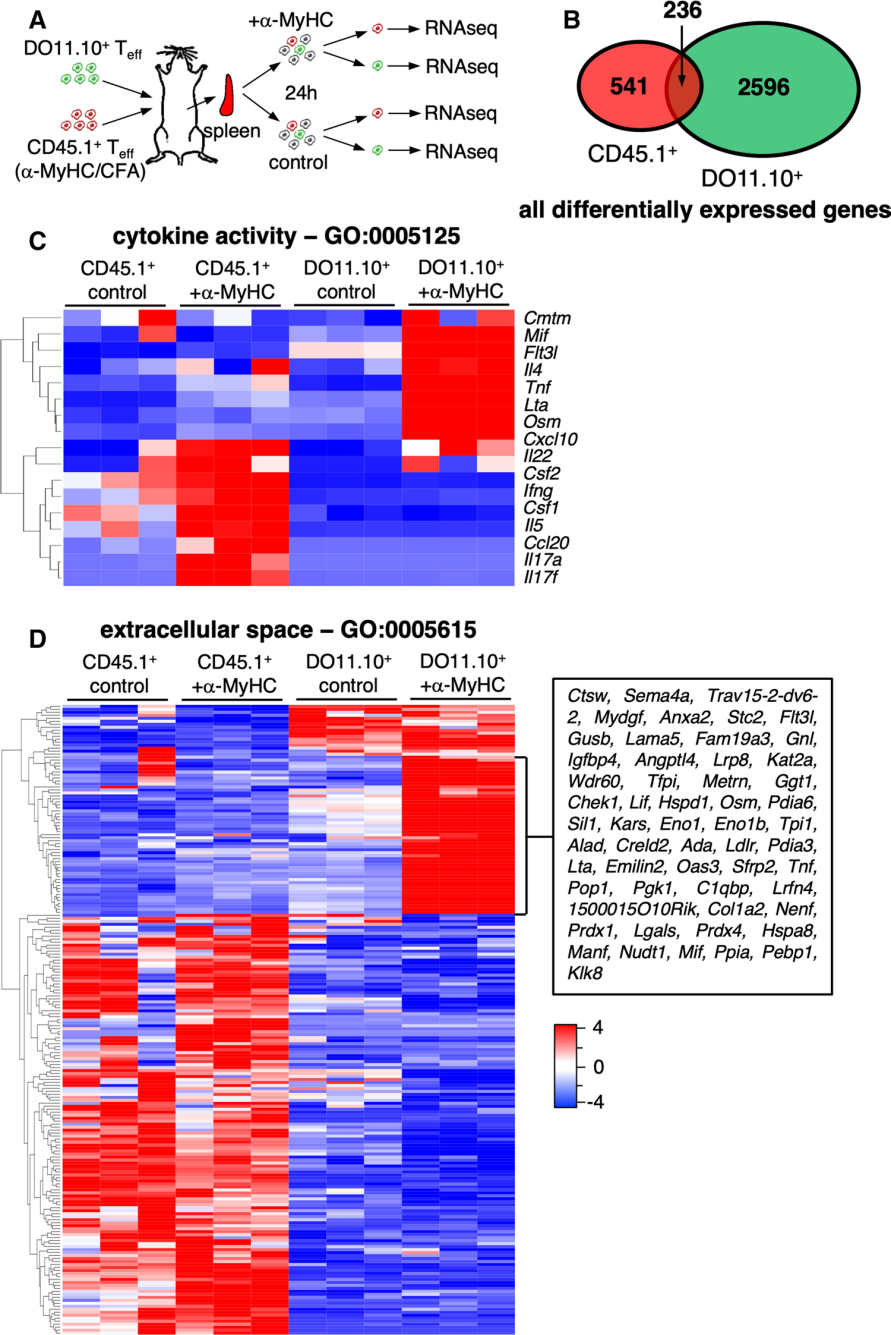
Transcriptomic analysis of heart-specific and heart non-specific *T*_eff_ cells. CD45.1^+^ *T*_eff_ (3 × 10^6^) and DO11.10^+^ *T*_eff_ (3 × 10^6^) cells were co-delivered into sub-lethally irradiated BALB/c recipients. 10 days later, the whole splenocytes were isolated, stimulated with α-MyHC (or kept unstimulated for controls) for 24 h and CD45.1^+^ and DO11.10^+^ CD4^+^ cells were sorted for the whole transcriptome sequencing (RNAseq). Panel **a** illustrates the experimental setup. The number and the overlap of all differentially expressed genes (*p *< 0.05, log2ratio > 0.5 or < −0.5) in response to α-MyHC in CD45.1^+^ *T*_eff_ and in DO11.10^+^ *T*_eff_ is shown in panel **b**. Panel **c** shows heatmap of genes of the cytokine activity GO:0005125 category up-regulated (*p *< 0.05, log2ratio > 0.75) in CD45.1^+^ *T*_eff_ and/or DO11.10^+^ *T*_eff_ in response to α-MyHC treatment. Panel **d** shows heatmap of genes of the extracellular space GO:0005615 category differentially expressed (*p *< 0.05, log2ratio > 0.75 or < − 0.75) between CD45.1^+^ *T*_eff_ and DO11.10^+^ *T*_eff_ under stimulatory (+α-MyHC) condition. Cells obtained from 3 independent mice were used for RNAseq. Donor CD45.1^+^ and DO11.10^+^ *T*_eff_ were generated as described in Fig. 2

To better characterize antigen-dependent and antigen-independent CD4^+^ T cell responses, splenocytes of mice adoptively co-transferred with CD45.1^+^ *T*_eff_ (obtained from α-MyHC/CFA immunized mice) and DO11.10^+^ *T*_eff_ were isolated and cultured with or without α-MyHC peptide for 24 h. Next, CD45.1^+^ *T*_eff_ and DO11.10^+^ *T*_eff_ were sorted and analyzed for the whole genome transcriptomics (Fig. 4a). The analysis showed differential response of CD45.1^+^ *T*_eff_ and DO11.10^+^ *T*_eff_ to α-MyHC stimulation (Fig. 4b). Antigen-dependent stimulation of CD45.1^+^ *T*_eff_ resulted in response with Th cytokines (*Il17a*, *Il17f, Ifng, Il5, Il22*), chemokines (*Ccl20, Cxcl10*) and hematopoietic growth factors (*Csf1, Csf2,* Fig. 4c). In contrast, antigen-independent response of DO11.10^+^ T_eff_ resulted in the up-regulation of inflammatory cytokines of TNF superfamily (*Tnf, Lta*), IL-6 family (*Osm, Lif*) and others (*Mif, Il4, Flt3l,* Fig. 4c).

### Heart non-specific *T*_eff_ do not affect myocarditis severity, but protect from post-inflammatory fibrosis and cardiac dysfunction

**Fig. 5:**
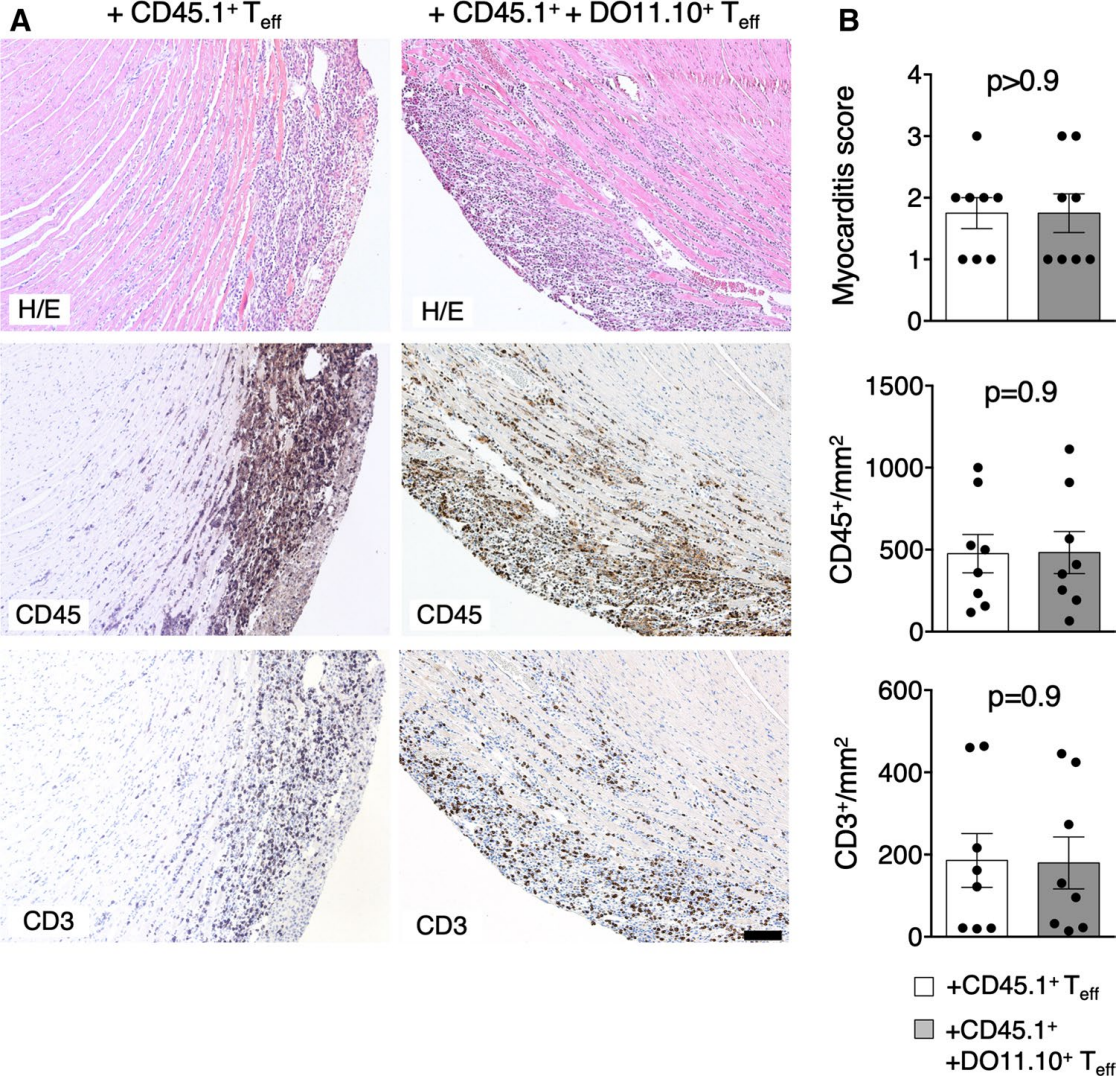
Heart non-specific *T*_eff_ do not affect myocarditis severity. Myocarditis-inducing CD45.1^+^ *T*_eff_ (4–6 × 10^6^) were injected with or without DO11.10^+^ *T*_eff_ (4–6 × 10^6^) into sub-lethally irradiated BALB/c recipients. Representative histology (top) and immunohistochemistry for CD45 (middle) and CD3 (bottom) in hearts of indicated recipients 10 days after adoptive transfer are shown in panel **a**. Scale bar = 100 μm. Myocarditis severity scores and quantification of heart-infiltrating CD3^+^ and CD45^+^ cells are presented in panel **b**. *p* values calculated with unpaired Student’s *t* test. Donor CD45.1^+^ and DO11.10^+^ *T*_eff_ were generated as described in Fig. 2

**Fig. 6:**
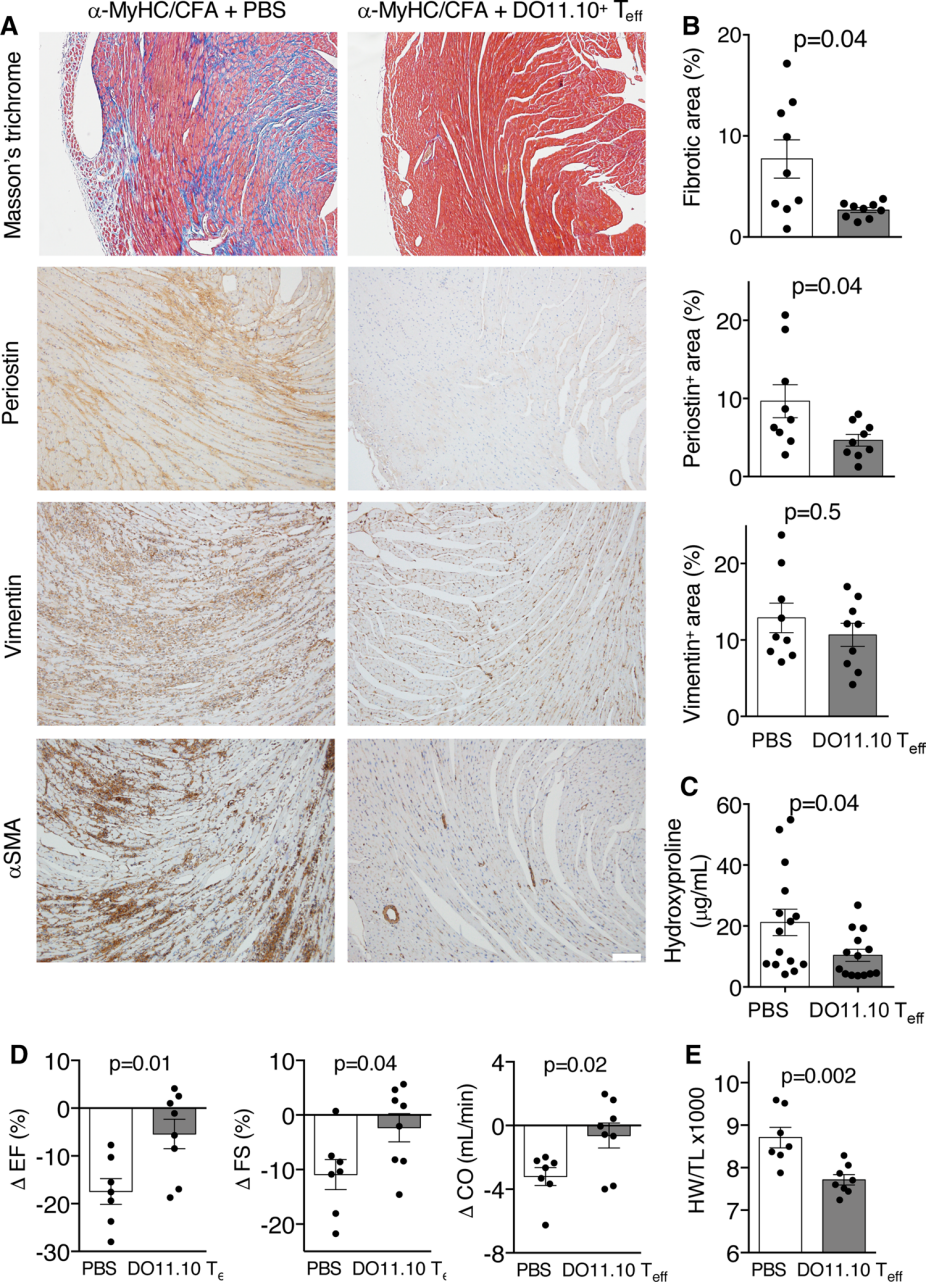
Heart non-specific *T*_eff_ prevents postinflammatory fibrosis and cardiac dysfunction. α-MyHC/CFA immunized BALB/c recipients received control solution (PBS) or DO11.10^+^ *T*_eff_ (4–5 × 10^6^) at days 17 and 20 of EAM and were analyzed for fibrotic and functional changes at day 40. Panel **a** shows representative Masson’s Trichrome staining and immunohistochemistry stainings for periostin, vimentin and αSMA in heart sections. Scale bar = 200 μm. Quantifications of the fibrotic and immunopositive areas are shown in panel **b**. *p* values calculated with the Mann–Whitney test. Panel **c** shows hydroxyproline content at day 40. Echocardiography measurements were performed in mice before immunization (d0) and at d40 of EAM. Panel **d** shows difference for d40 and d0 (Δ = d40–d0) for selected parameters. *EF* ejection fraction, *FS* fractional shortening, *CO* cardiac output. *p* values calculated with the Student’s *t* test. All echocardiographic parameters at d0 and d40 are available in the Suppl. Table 2. Analysis of heart weights at d40 is shown in panel **e**. *HW/TL* heart weight to tibial length. *p* values calculated with the Student’s *t* test. Donor DO11.10^+^ *T*_eff_ were generated as described in Fig. 2

To address functional consequences of differential CD4^+^ T cell responses, we analyzed heart sections of mice receiving α-MyHC-reactive CD45.1^+^ *T*_eff_ (obtained from α-MyHC/CFA immunized mice) with or without heart non-specific DO11.10^+^ *T*_eff_. Accumulation of heart non-specific DO11.10^+^ *T*_eff_ neither affected myocarditis severity scores, nor the total number of CD3^+^ and CD45^+^ infiltrates in cardiac sections (Fig. 5). Next, we addressed the impact of heart non-specific *T*_eff_ on cardiac fibrosis in α-MyHC/CFA-immunized BALB/c mice. Immunized mice received two doses of either antigen-activated DO11.10^+^ or TCR-M *T*_eff_, or non-activated DO11.10^+^ *T*_n_ shortly before the onset of myocardial fibrosis at d17 and d20. Both DO11.10^+^ and TCR-M *T*_eff_ infiltrated the inflamed myocardium (Supp. Fig. 4). Injection of DO11.10^+^ *T*_eff_ protected the heart from post-inflammatory fibrosis at later stage (day 40), as indicated by Masons Trichrome staining (Fig. 6a, b), reduced hydroxyproline content (Fig. 6c), expression of profibrotic genes (Suppl. Fig. 5) and the amount of αSMA-positive and periostin-postive myofibroblasts (Fig. 6a, b). Instead, injection of TCR-M *T*_eff_ or DO11.10^+^ *T*_n_ did not affect post-inflammatory fibrosis (Suppl. Fig. 6). Next, we addressed whether DO11.10^+^ *T*_eff_ cells can affect cardiac function. As expected, echocardiography analysis showed significantly compromised cardiac function in α-MyHC/CFA-immunized mice at day 40 with ejection fraction reduced from 58% before immunisation to 40% at d40 and fractional shortening from 29% before immunisation to 18% at d40. Treatment with DO11.10^+^ *T*_eff_ cells prevented development of cardiac dysfunction in most animals (Fig. 6d and Suppl. Fig. 7). This better cardiac functions in mice receiving DO11.10^+^ *T*_eff_ cells were paralleled by a lower heart weights comparing with α-MyHC/CFA-immunized mice treated with PBS (Fig. 6e). All these data suggest that heart non-specific *T*_eff_ cells can prevent development of DCM phenotype in the EAM model.

### Bystander activation of *T*_eff_ suppress myofibroblast phenotype of mouse and human cardiac fibroblasts

The transcriptomic analysis showed that in contrast to heart-specific CD45.1^+^ CD4^+^ T cells, unstimulated DO11.10^+^ *T*_eff_ expressed low levels of extracellular molecules. In stimulatory condition (+α-MyHC), however, a set of 56 genes encoding extracellular factors became specifically upregulated in heart non-specific DO11.10^+^ *T*_eff_, but not in α-MyHC-reactive CD45.1^+^ *T*_eff_ (Fig. 4d). This suggested that antigen-independent stimulation of *T*_eff_ might trigger production of certain antifibrotic factors. To address this option, we co-cultured DO11.10^+^ *T*_eff_ with mouse cardiac fibroblasts in the presence or absence of IL-2, IL-7, IL-15 and IL-21 (further called γc-cytokines). Analysis of profibrotic markers in cardiac fibroblasts showed that DO11.10^+^ *T*_eff_ stimulated with γc-cytokines effectively suppressed expression of profibrotic genes *Acta2*, *Col1a1* and *Fn1* and reduced formation of αSMA fibers (Supp. Fig. 8).

**Fig. 7:**
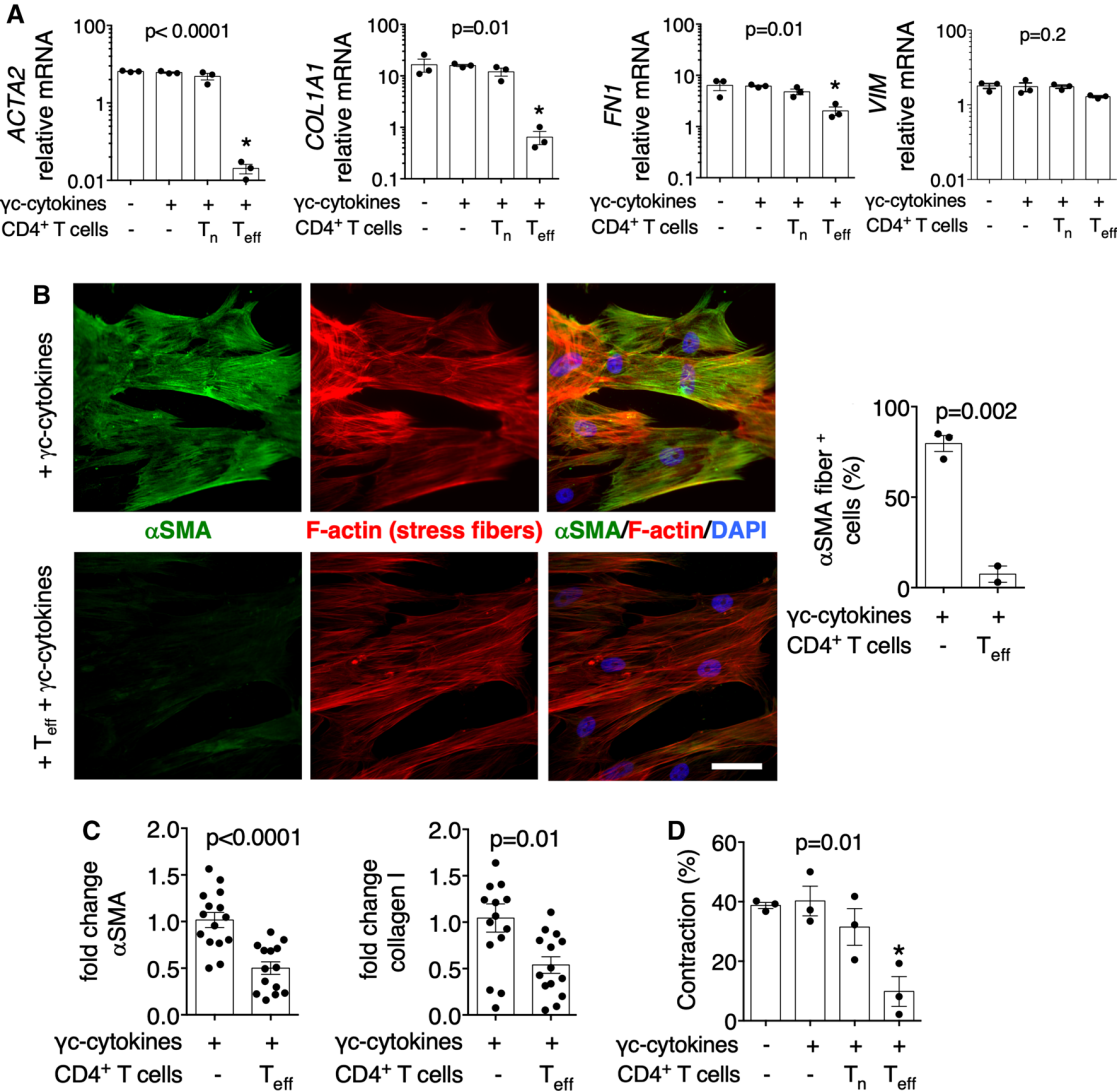
Human *T*_eff_ stimulated with γc-cytokines suppress myofibroblast signature of human cardiac fibroblasts. Human cardiac fibroblasts were co-cultured with sorted human *T*_n_ and *T*_eff_ in the presence of IL-2, IL-7, IL-5 and IL-21 (γc-cytokines) for 7 days (human *T*_n_ and *T*_eff_ were isolated as shown in Supp. Fig. 7). Panel **a** shows gene expression levels (normalized to *GAPDH)* in cardiac fibroblasts at day 7 at indicated conditions. Data are representative of three independent experiments, *p* values calculated with one-way ANOVA, * *p *< 0.05 the Dunnett post hoc test compared to fibroblasts with γc-cytokines. Panel **b** shows representative immunofluorescence for αSMA (green) and F-actin (phalloidin staining, red) and quantification of αSMA-positive cardiac fibroblasts cultured for 7 days in presence of γc-cytokines with or without human *T*_eff_. Data are representative of three independent experiments, *p* value calculated with unpaired Student’s *t*-test. Scale bar = 50 μm. Panel **c** shows relative intracellular levels of αSMA and collagen I in cardiac fibroblasts in presence of γc-cytokines and *T*_eff_ measured with flow cytometry. Data are shown as fold change from three independent experiments, *p* values calculated with unpaired Student’s *t*-test. Panel **d** shows contraction of cardiac fibroblasts in 3D collagen matrix after 5 days. Cardiac fibroblasts were pretreated with γc-cytokine-stimulated *T*_n_ or *T*_eff_ for 7 days. Data are representative of two independent experiments, *p* values calculated with one-way ANOVA, * *p *< 0.05 the Dunnett post hoc test compared to fibroblasts with γc-cytokines. *ACTA2* alpha smooth muscle actin, *COL1A1* collagen I, *FN1* fibronectin, *VIM* vimentin

Next, we addressed, whether or not, human *T*_eff_ can affect the myofibroblast phenotype of human cardiac fibroblasts. A panel of phenotypic markers (CD45RO, CD45RA, CCR7, CD27) allows identification of CD4^+^ T cell subsets in humans (Supp. Fig. 9A). Using these markers, we sorted *T*_n_ (CD45RO^−^CD45RA^+^CCR7^+^CD27^+^) and *T*_eff_ (CD45RO^+^CD45RA^−^CCR7^−^CD27^−^) from human peripheral blood and analyzed their proliferation in vitro. In contrast to *T*_n_ cells, human *T*_eff_ showed some proliferation in response to γc-cytokine stimulation (Supp. Fig. 9B). In co-cultures with human cardiac fibroblasts, only *T*_eff_ stimulated with γc-cytokines down-regulated expression of profibrotic genes *ACTA2*, *COL1A1*, and *FN1* in fibroblasts (Fig. 7a). Furthermore, in co-cultures with *T*_eff_, cardiac fibroblasts showed less αSMA-positive fibers (Fig. 7b). The reduced αSMA and collagen-I protein content was confirmed by intracellular flow cytometry analysis (Fig. 7c). Since αSMA is implicated in the contractile function of fibroblasts, we observed impaired contraction of cardiac fibroblasts upon pre-treatment with γc-cytokine-stimulated *T*_eff_ (Fig. 7d). Suppression of myofibroblast signature in cardiac fibroblasts by *T*_eff_ was, however, not associated with changes in proliferation or apoptosis (Supp. Fig. 10). Our data suggested that the antifibrotic effect of *T*_eff_ required stimulation with γc-cytokines.

## Discussion

Observations from animal models have pointed to a critical role of CD4^+^ T cells and heart-specific autoimmunity in the development of myocarditis and cardiac fibrosis, however, surprisingly little experimental data addressed migration and expansion of autoreactive CD4^+^ T cells in the EAM model. In α-MyHC/CFA immunized mice, essentially only α-MyHC-reactive T cells could be activated with their antigen and turn into *T*_eff_ phenotype. Our mouse data confirmed that α-MyHC-reactive *T*_eff_ cells efficiently expand and accumulate in the inflamed cardiac tissue. Furthermore, we demonstrated *T*_eff_ phenotype of heart infiltrating CD4^+^ T cells also in human myocarditis. Animals housed under high hygiene standards lack, however, other antigen-activated *T*_eff_ cells, which are commonly present in humans due to exposure to various infectious agents. To address migratory properties of heart non-specific *T*_eff_, we used a T cell adoptive transfer model of DO11.10^+^ CD4^+^ T cells activated in vitro with OVA antigen and found that *T*_eff_ cardiotropism was not restricted to heart-specific CD4^+^ T cells. It seems that expression of specific homing molecules, rather than antigen-specificity primary controls migration of CD4^+^ T cells into the cardiac tissue. In fact, such cardiotropic signature has been recently suggested [23]. In our model, we observed more efficient accumulation of heart-specific CD4^+^ T cells during the early phase of inflammation, while heart non-specific CD4^+^ T cells were more prevalent at later phases. This differential kinetics could be the result of differential cell expansion/survival in the periphery and/or differential expression of homing molecules by these two types of CD4^+^ T cells.

Antigen-specific responses of α-MyHC-reactive CD4^+^ T cells are crucial for myocarditis induction. Our results pointed to Th1/Th17 skewed antigen-dependent response of heart-specific *T*_eff_. On the one hand, Th1 cytokines are considered to be involved in the myocarditis induction at a very early stage [37]. Th17 polarized responses, on the other hand, have been associated with worsened myocarditis outcome due to increased cardiac fibrosis and heart failure in both, EAM and human patients [2, 29, 31]. These data might suggest that myocardial fibrosis and DCM phenotype after α-MyHC/CFA immunization is a direct consequence of the Th1/Th17 polarisation of α-MyHC-reactive CD4^+^ T cells. Furthermore, adoptive transfer experiments demonstrated that heart-specific CD4^+^ T cell mediated myocarditis induction paralleled a strong bystander activation of heart non-specific CD4^+^ T cells. Such antigen-independent expansion of activated *T*_eff_ has been described in the context of viral infections [13], in breast cancer [17] and after vaccination [10]. Insights from other disease models point to various innate stimuli [18, 32] and γc-cytokines [12, 33] as potential mediators. We assume that in myocarditis, mainly γc-cytokines produced by heart-specific CD4^+^ T cells promote the antigen-independent response of heart non-specific CD4^+^ T cells.

In our model heart non-specific *T*_eff_ effectively competed with heart-specific *T*_eff_ and eventually partially replaced them in the inflamed myocardium at later stages of disease. Our data showed that heart non-specific *T*_eff_ produced a distinct set of cytokines compared to heart-specific *T*_eff_. This is not surprising, considering that heart-specific *T*_eff_ responded to antigen (α-MyHC), while heart non-specific *T*_eff_ showed antigen-independent response only. Interestingly, heart non-specific *T*_eff_ expressed high levels of pro-inflammatory cytokines, such as *Tnf*, *Lta* or *Mif*. This result could explain why heart non-specific *T*_eff_ did not affect the severity of the acute cardiac inflammatory response.

In contrast, adoptive transfer of heart non-specific *T*_eff_ successfully prevented development of post-inflammatory fibrosis in the EAM model. We found that bystander activation of the heart non-specific *T*_eff_ induced production of surprisingly high number of extracellular molecules. Some of these factors might be antifibrotic and thus actively inhibit fibrotic processes. *Sfrp2* represents an example of such antifibrotic agent, which was specifically expressed by activated heart non-specific *T*_eff_. We have recently demonstrated that systemic administration of sFRP2 during acute phase of myocarditis prevented development of fibrotic changes in the EAM model by blocking profibrotic Wnt signalling [5]. The in vitro data have also pointed to the secretion of antifibrotic factors by mouse and human *T*_eff_ activated in antigen-independent manner. Furthermore, heart non-specific *T*_eff_ by replacing heart-specific *T*_eff_ could reduce cardiac levels of profibrotic cytokines, such as IL-17A, in the inflamed heart. We consider these two mechanisms being responsible for antifibrotic effect of the heart non-specific *T*_eff_ (Supp. Fig. 11). Noteworthy, the analysis of cytokine and extracellular factors production by heart-specific and heart non-specific *T*_eff_ was performed on restimulated splenic cells only. Lack of data obtained from the heart-infiltrating *T*_eff_ is a major limitation of this study. We did not perform such analysis due to technical difficulties.

Cardiac fibrosis is a key driver of DCM [6]. Accordingly, in the EAM model, development of cardiac fibrosis was associated with impaired cardiac function and increased heart size [4]. Our data confirmed that function of fibrotic heart are significantly compromised in the α-MyHC/CFA EAM model (ejection fraction reduced from 58% before immunisation to 40% at d40). It is, therefore, not surprising that antifibrotic effect of heart non-specific *T*_eff_ protected cardiac function and development of DCM phenotype. It should be noted that interplay between cardiac inflammation and fibrosis is of high relevance, not only in myocarditis, but also in other heart diseases [1]. These results further stress the importance of antifibrotic therapies for the positive outcome of myocarditis. A number of antifibrotic agents successfully prevented development of DCM phenotype in animal models [4, 5, 20, 25].

In contrast to the EAM model, heart-specific T cells are usually not the primary inducers of myocarditis in humans [7, 22]. Instead, viruses and parasites represent the most common triggers of lymphocytic infiltration in the myocardium and heart-specific autoimmunity is predominantly associated with the disease progression [3, 8]. By shedding light on the importance of antigen-independent responses, our data provide a link between insights from animal models and observations in humans. Accordingly, our findings can potentially explain the difficulties in identifying heart-specific CD4^+^ T cells in human myocarditis. We suggest that inflammation-driven, antigen-independent migration and proliferation of *T*_eff_ could account for extensive cardiac accumulation of heart non-specific CD4^+^ T cells. So far, there are no concrete data on the numbers of heart-reactive CD4^+^ T cells in human myocarditis, but considering that heart-specific CD4^+^ T cells are rather infrequent in humans, we hypothesize that a significant proportion of heart-infiltrating CD4^+^ T cells in myocarditis patients do not react against cardiac antigens. From this point of view, and given the antifibrotic and cardioprotective properties of heart non-specific *T*_eff_, significant infiltrations with CD4^+^ T lymphocytes could even indicate favourable outcome in myocarditis. Indeed, most patients with lymphocytic myocarditis recover, while only some develop DCM phenotype [11, 28]. The observation that chronic inflammation and long-term antigen activation alters *T*_eff_ responses [16] supports our idea that a bystander activation of CD4^+^ T cell protects myocarditis patients from progression to heart failure.

So far, investigations addressing the role of CD4^+^ T cells in myocarditis focused on heart-specific autoimmunity. Our data underline potential importance of antigen-independent CD4^+^ T cell responses in the progression from myocarditis to DCM and attribute them a cardioprotective role. Individual differences in the capability of the immune system to express such functions could explain variable clinical outcomes of lymphocytic myocarditis. The relevance of our findings has to be validated in human patients, since EAM model only partially mimics the complexity of naturally occurring myocarditis. At the same time, further research is needed to elucidate molecular mechanisms and to enable translation of these findings into appropriate therapeutic approaches.

## Electronic supplementary material

Below is the link to the electronic supplementary material.

Supplementary material 1 (PDF 2188 kb)

Supplementary material 2 (DOCX 59 kb)

## Abbreviations

EAM: Experimental autoimmune myocarditis
DCM: Dilated cardiomyopathy
α-MyHC: Alpha-myosin heavy chain
CFA: Complete Freud’s adjuvant
*T*_n_/*T*_eff_: Naïve/effector CD4^+^ T cell
TCR: T cell receptor
APC: Antigen presenting cell
γc-cytokines: Common gamma cytokines
IL: Interleukin
αSMA: α-Smooth-muscle actin
LN: Lymph node
OVA: Ovalbumin
Th: CD4^+^ T helper cell
