# Supplementary material for: Heart non-specific effector CD4+ T cells protect from postinflammatory fibrosis and cardiac dysfunction in experimental autoimmune myocarditis
Source: Basic Res Cardiol. 2019 Dec 20;115(1):6. doi: 10.1007/s00395-019-0766-6 (PMC6925074; doi:10.1007/s00395-019-0766-6)
Supplement: Supplementary file 1 — Supplementary material 1 (PDF 2188 kb) [file 395_2019_766_MOESM1_ESM.pdf]

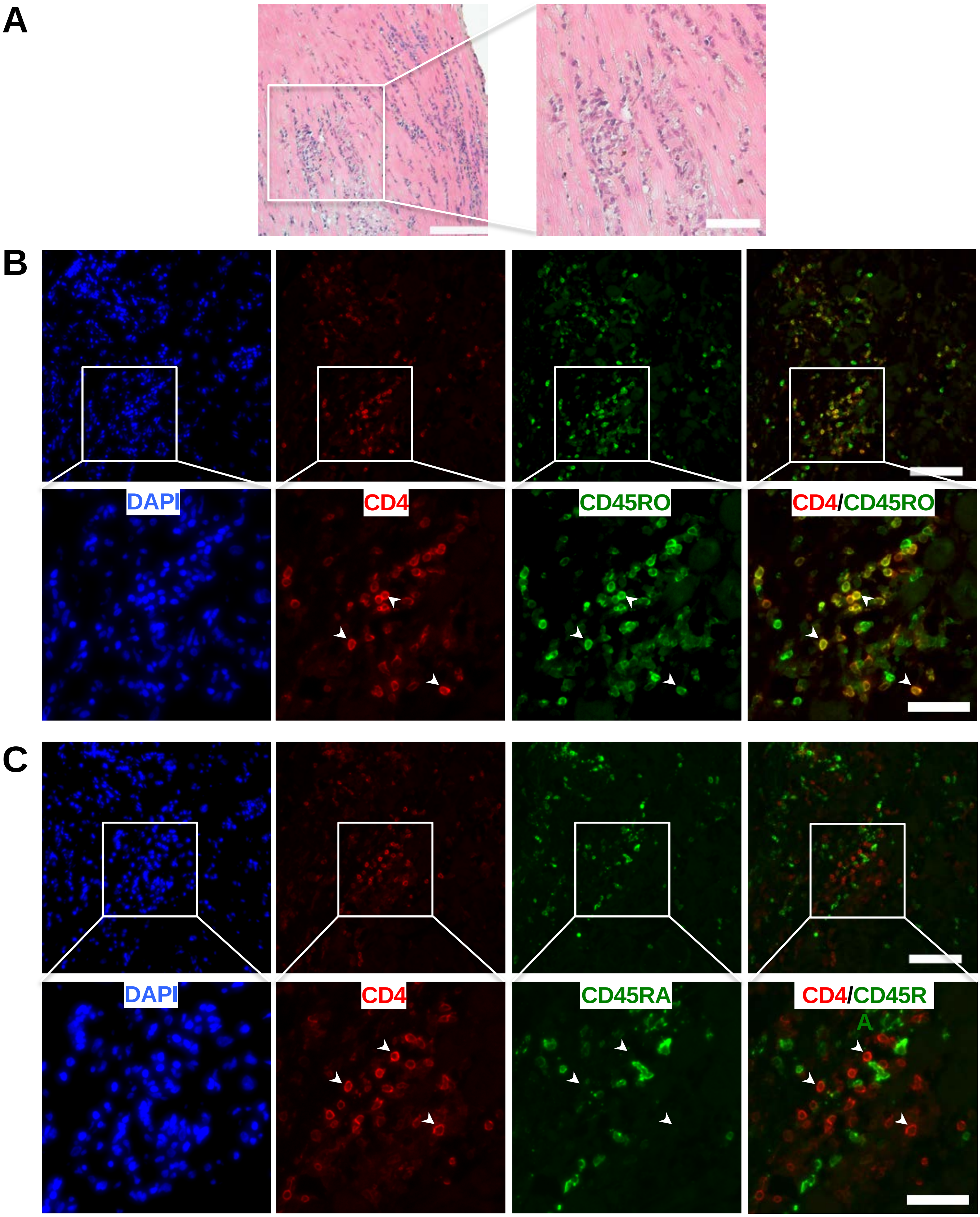

Supplementary Figure 1. T_{eff} phenotype of heart-infiltrating $CD4^+$ T cells in lymphocytic myocarditis. Panel (A) shows representative histology of human heart tissue with acute lymphocytic myocarditis. Panels (B, C) present immunofluorescent staining of inflammatory infiltrates in lymphocytic myocarditis. Panel (B) shows staining for CD4 (red) and T cell activation marker CD45RO (green). Arrows indicate double positive ($CD4^+CD45RO^+$) T_{eff}. Panel (C) shows staining for CD4 (red) and naïve/resting cell marker CD45RA (green). Arrows indicate $CD4^+$ cells negative for CD45RA antigen. Scale bars 100 µm; enlarged images 50 µm.

Supplementary Figure 1

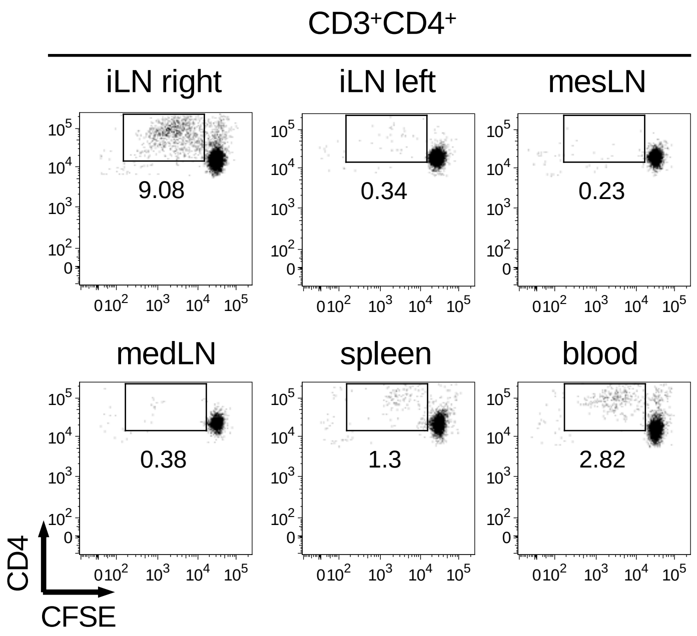

Supplementary Figure 2. Local activation of heart-reactive CD4+ T cells in α-MyHC/CFA-induced EAM. BALB/c mice received subcutaneous injection of α-MyHC/CFA into the right inguinal region as shown in Fig. 1A. CD4+ T cells isolated from indicated organs 5 days after immunization were stimulated for 3 days with α-MyHC peptide in presence of APCs. Proliferation was measured based on CFSE dye dilution in viable CD3+CD4+ cell population (B). Data are representative of 3 independent experiments. iLN – inguinal lymph node; mesLN – mesenteric lymph node; medLN – mediastinal lymph node.

Supplementary Figure 2

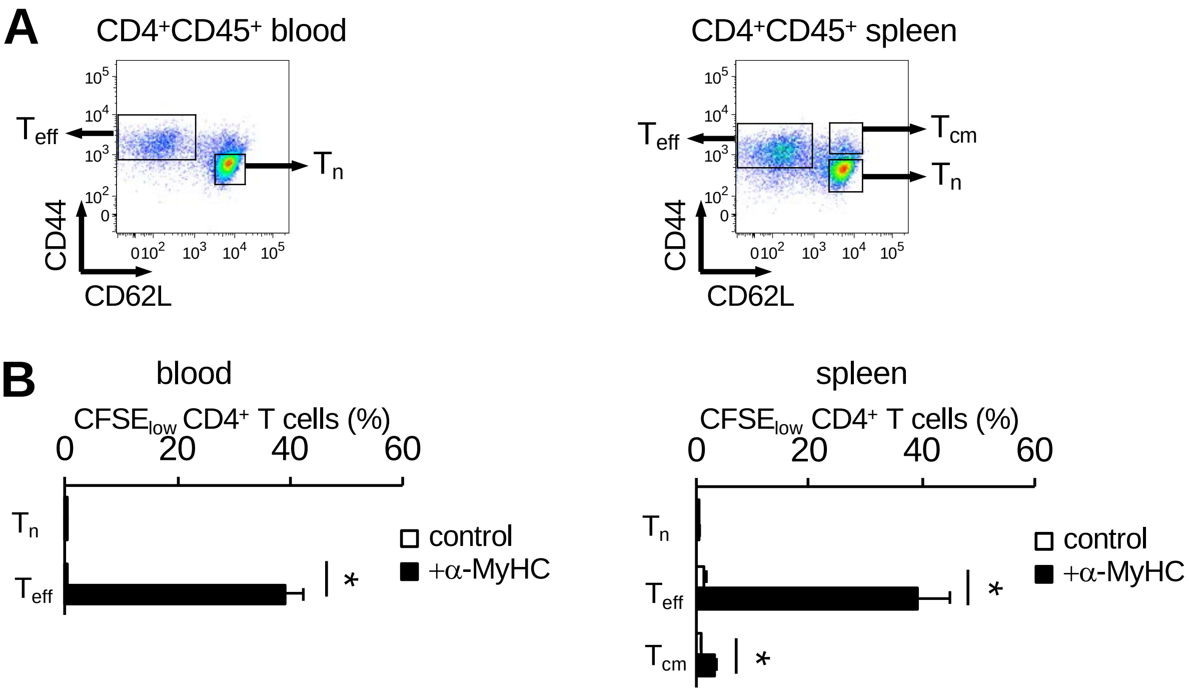

Supplementary Figure 3. Heart-reactive T_{eff} circulate in the bloodstream before onset of myocarditis in α-MyHC/CFA immunized mice. BALB/c mice were immunized with α-MyHC/CFA. Panel (A) shows the gating strategy for effector (T_{eff}), central memory (T_{cm}) and naïve (T_n) subpopulations from viable $CD4^+CD45^+$ cells isolated from blood (left) and spleen (right) 7 days after immunization (before myocarditis onset). Cells were sorted, labeled with CFSE and stimulated with α-MyHC peptide for 3 days in presence of APCs. Proliferation (CFSE dye dilution) of viable $CD3^+CD4^+$ cells is shown in (B); n=3, data are representative of 3 independent experiments, * $p<0.05$ unpaired Student's t-test.

Supplementary Figure 3

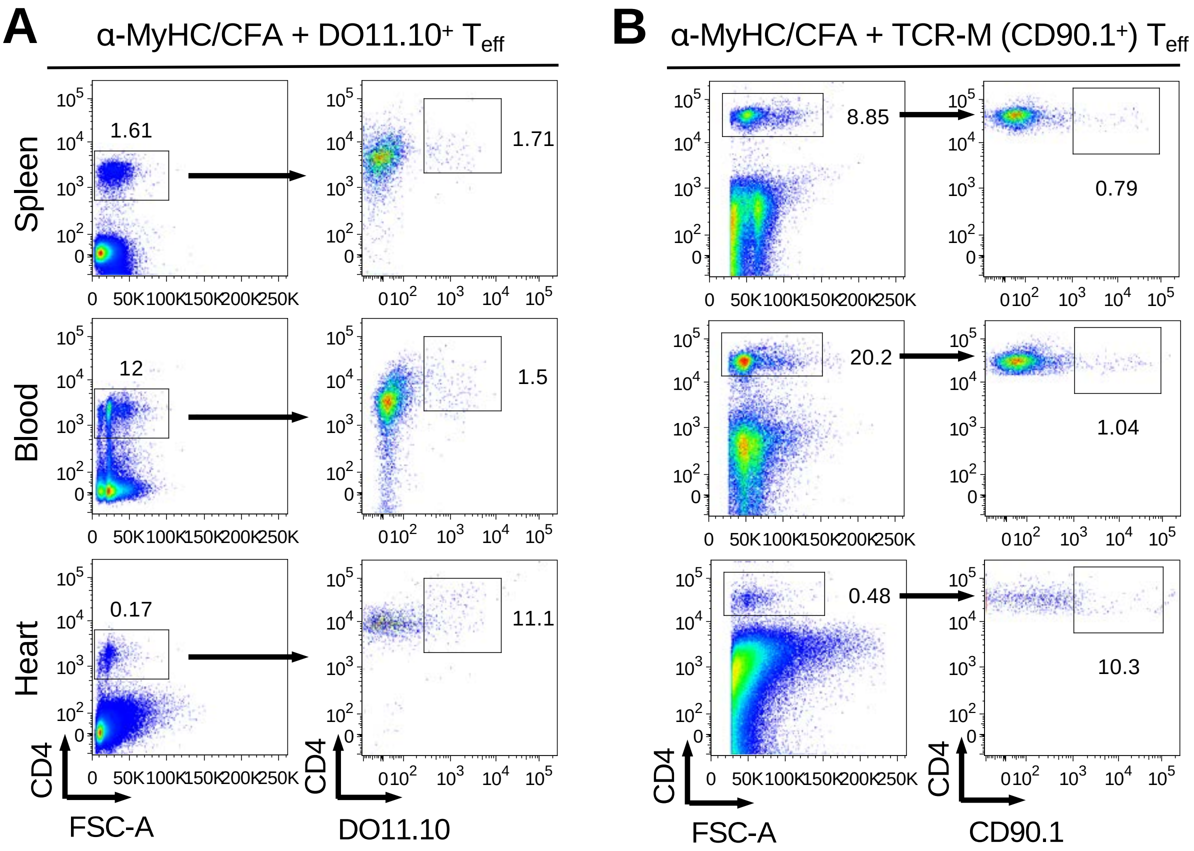

Supplementary Figure 4. Intravenously injected DO11.10⁺ and TCR-M T_{eff} infiltrate the heart of α-MyHC/CFA immunized mice. DO11.10⁺ T_{eff} ($5x10^6$) or CD90.1⁺ TCR-M T_{eff} ($3.5x10^6$) were injected into α-MyHC/CFA immunized BALB/c mice at day 17 of EAM (active myocarditis). Infiltration of injected T_{eff} in indicated organs at day 20 is shown for DO11.10⁺ (A) and TCR-M (B) cells. Arrows show the gating strategy and numbers indicate percentage of cells in the adjacent gate. Data are representative of 6 mice for (A) and one mouse for (B). Donor DO11.10⁺ and TCR-M T_{eff} were generated as described in Fig. 2.

Supplementary Figure 4

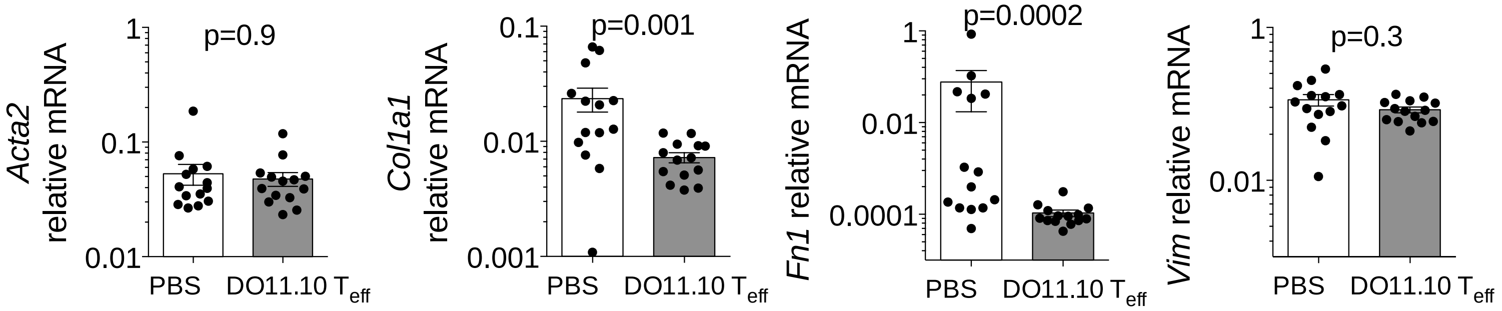

Supplementary Figure 5. Heart non-specific Teff prevent postinflammatory fibrosis. α-MyHC/CFA immunized BALB/c recipients received control solution (PBS) or DO11.10+ Teff (4-5x106) at days 17 and 20 of EAM and were analyzed for fibrotic changes at day 40. Relative expression of profibrotic genes in cardiac tissues are shown. p values calculated with the Mann-Whitney test. Donor DO11.10+ Teff were generated as described in Fig. 2. Acta2 – alpha smooth muscle actin; Col1a1 – collagen I; Fn1 – fibronectin; Vim – vimentin.

Supplementary Figure 5

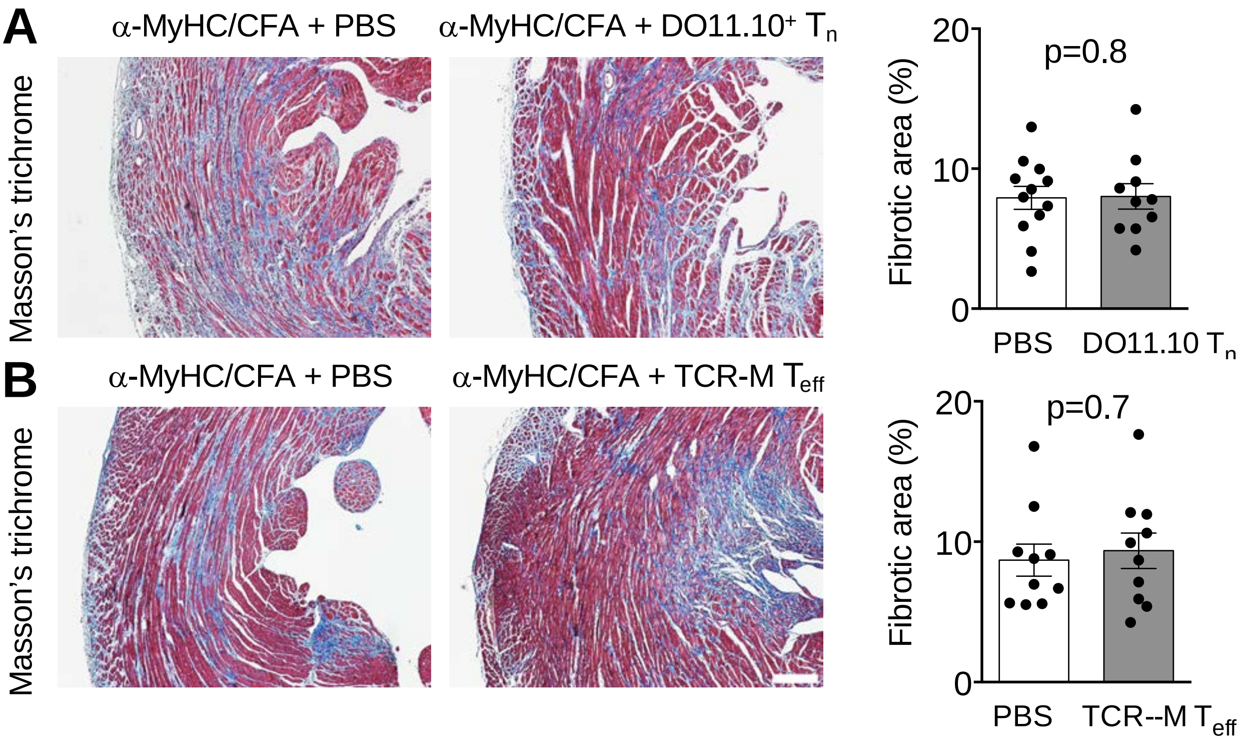

Supplementary Figure 6. α-MyHC-specific T_{eff} and DO11.10$^+$ T_n do not influence postinflammatory fibrotic remodelling. α-MyHC/CFA immunized BALB/c recipients received control solution (PBS) or either DO11.10$^+$ T_n (4x10^6) or TCR-M T_{eff} (3-4x10^6) at days 17 and 20 of EAM. Panels (A, B) show representative Masson's Trichrome staining for fibrosis in hearts of indicated recipients 40 days after immunization. Scale bar 200 µm. Quantification of the fibrotic areas is shown on the right; n=10-12, *p*-values calculated with the Mann-Whitney test. Donor TCR-M T_{eff} were generated as described in Fig. 2. DO11.10$^+$ T_n cells were sorted from unstimulated spleens.

Supplementary Figure 6

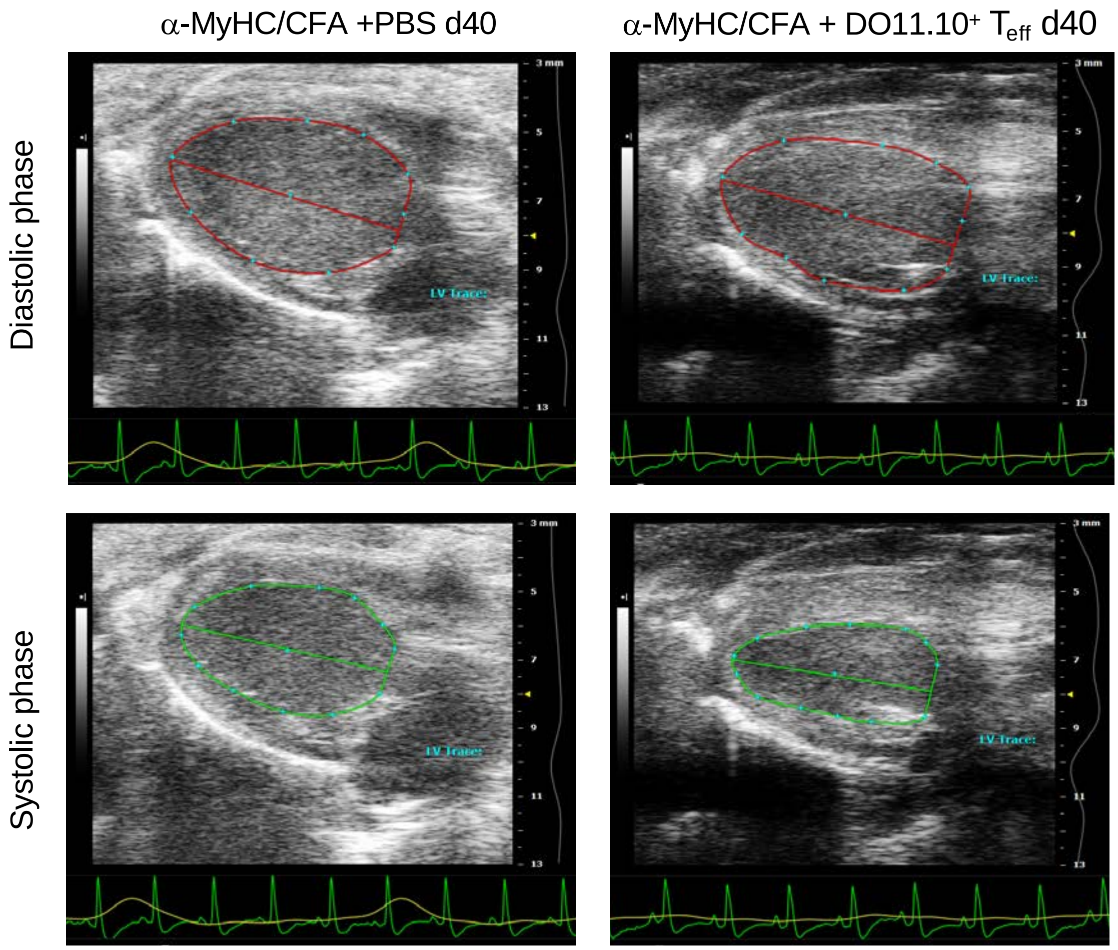

Supplementary Figure 7. Echocardiography Representative echocardiography of left ventricles at systolic and diastolic phase of α-MyHC/CFA immunized BALB/c recipients receiving control solution (PBS) or DO11.10+ Teff (4-5x106) at days 17 and 20 of EAM. Echocardiography was performed at day 40. Green and red lines indicate left ventricular lumens at systolic and diastolic phase respectively.

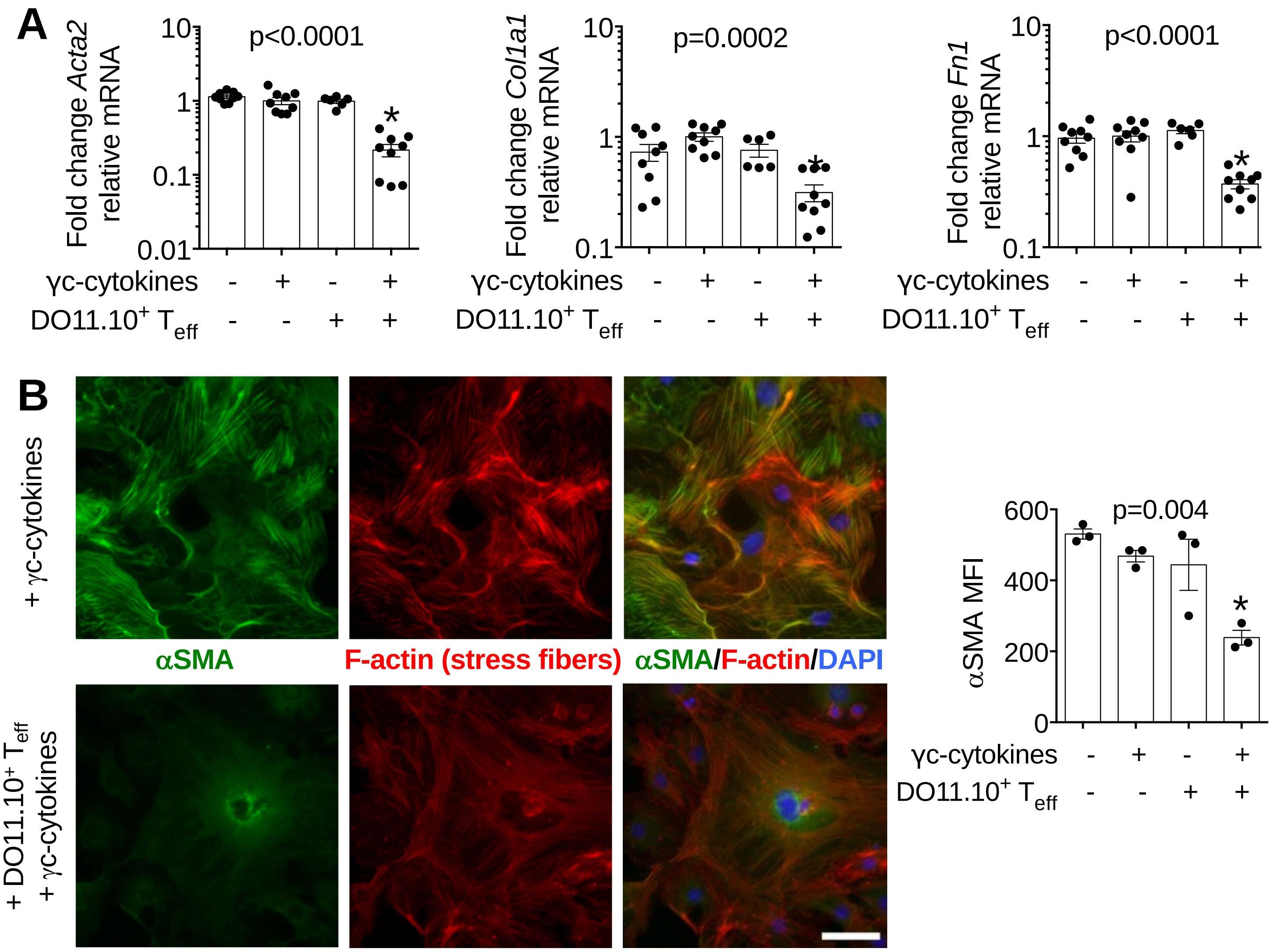

Supplementary Figure 8. T_{eff} stimulated with γc-cytokines supress the myofibroblast signature of mouse cardiac fibroblasts. Panel (A) shows gene expression levels (normalized to *gapdh*) in cardiac fibroblasts 24h after co-culture with or without DO11.10^{+} T_{eff} in presence or absence of IL-2, IL-7, IL-5 and IL-21 (γc-cytokines); n=9, data are shown as fold change from 3 independent experiments performed in triplicates, *p*-values calculated with one-way ANOVA, * $p<0.05$ the Dunnett *post-hoc* test compared to fibroblasts with γc-cytokines. Panel (B) shows representative immunofluorescence for αSMA (green) and F-actin (phalloidin staining, red) in cardiac fibroblasts cultured for 5 days in presence of γc-cytokines with or without DO11.10^{+} T_{eff} and quantification of αSMA mean fluorescent intensity (MFI); n=3, data are representative of 3 independent experiments, *p*-values calculated with one-way ANOVA, * $p<0.05$ the Dunnett *post-hoc* test compared to fibroblasts with γc-cytokines. Scale bar 50 µm. *Acta2* – alpha smooth muscle actin; *Col1a1* – collagen I; *Fn1* – fibronectin.

Supplementary Figure 8

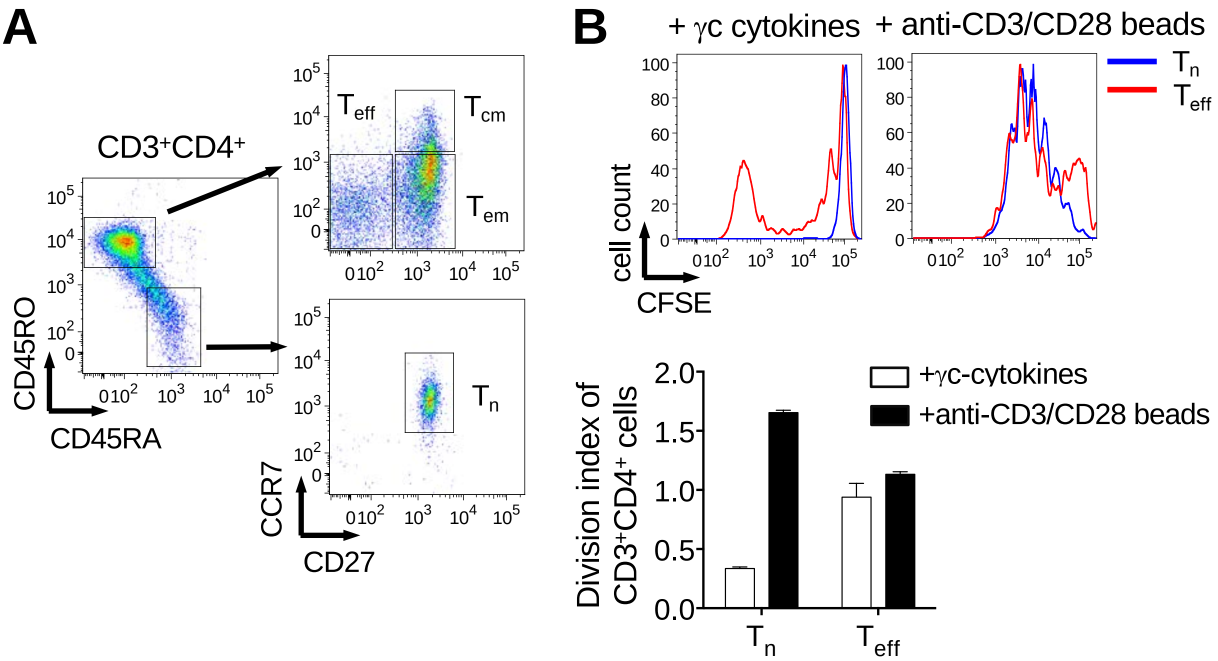

Supplementary Figure 9. Antigen-dependent and antigen-independent proliferation of human T_n and T_{eff}. T_n and T_{eff} were FACSorted from viable $CD4^+$ human peripheral blood cells based on phenotypic markers (CD45RA, CD45RO, CCR7, CD27) as shown in (A). Arrows indicate gating strategies. Proliferation of T_n and T_{eff} in response to anti-CD3/CD28 beads (5 days) and γc-cytokine stimulation (IL-2 IL-7 IL-5 IL-21; 7 days) analyzed as CFSE dye dilution is presented as division index in (B); n=3-5, data are representative of 2 independent experiments.

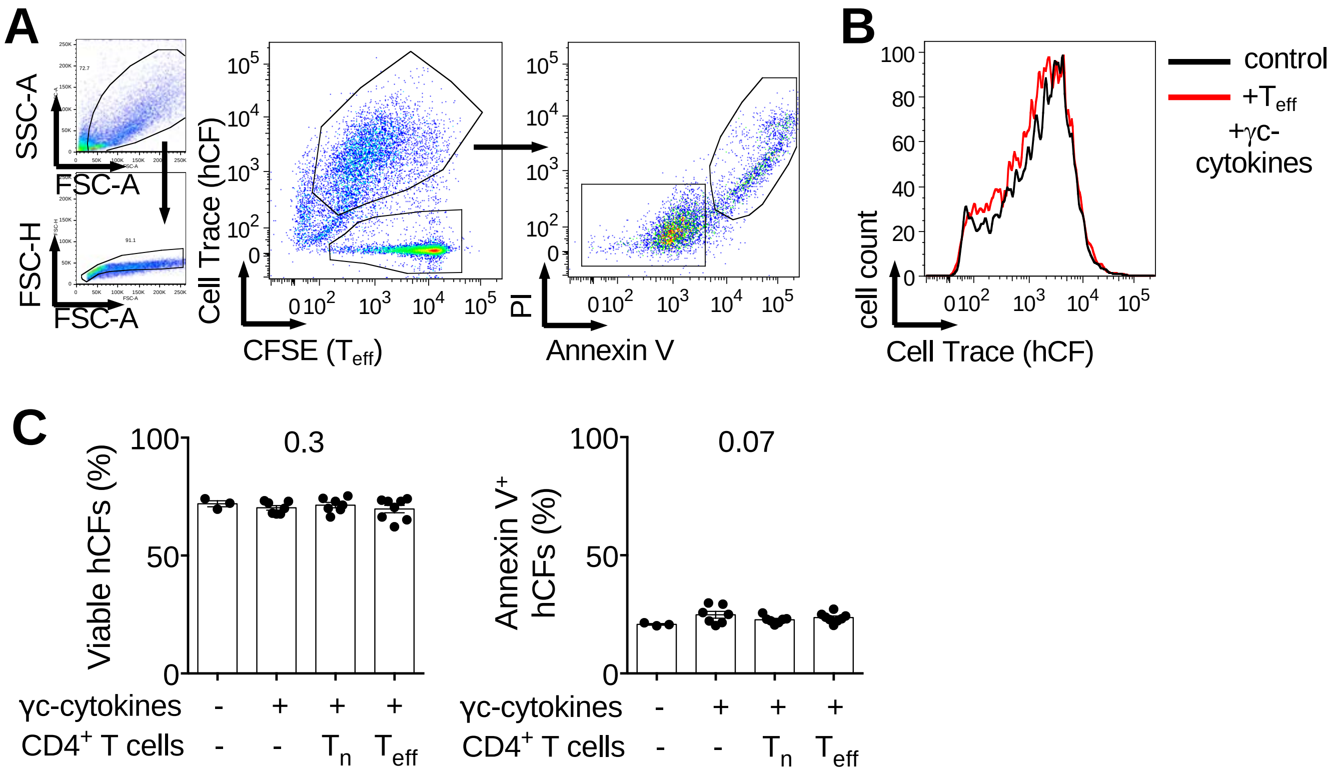

Supplementary Figure 10. Viability of human cardiac fibroblasts in co-cultures with T_{eff}. Human T_n and T_{eff} were isolated as shown in Supp. Fig. 7 and stained with CFSE. Human cardiac fibroblasts were stained with Cell Trace dye, co-cultured with T_n or T_{eff} for 7 days and analysed by flow cytometry. Panel (A) shows the gating strategy for analysis of cell viability in cardiac fibroblasts. Gates indicate viable cells (defined as Annexin V^- PI^-) and apoptotic cells (defined as Annexin V^+). Panel (B) shows dilution of Cell Trace dye, which indicates cardiac fibroblast proliferation. Panel (C) shows quantification of viable and apoptotic cardiac fibroblasts; n=5, *p*-values calculated with one-way ANOVA; data are representative of 2 independent experiments. *ACTA2* – alpha smooth muscle actin; *COL1A1* – collagen I; *FN1* – fibronectin; hCF – human cardiac fibroblast.

Supplementary Figure 10

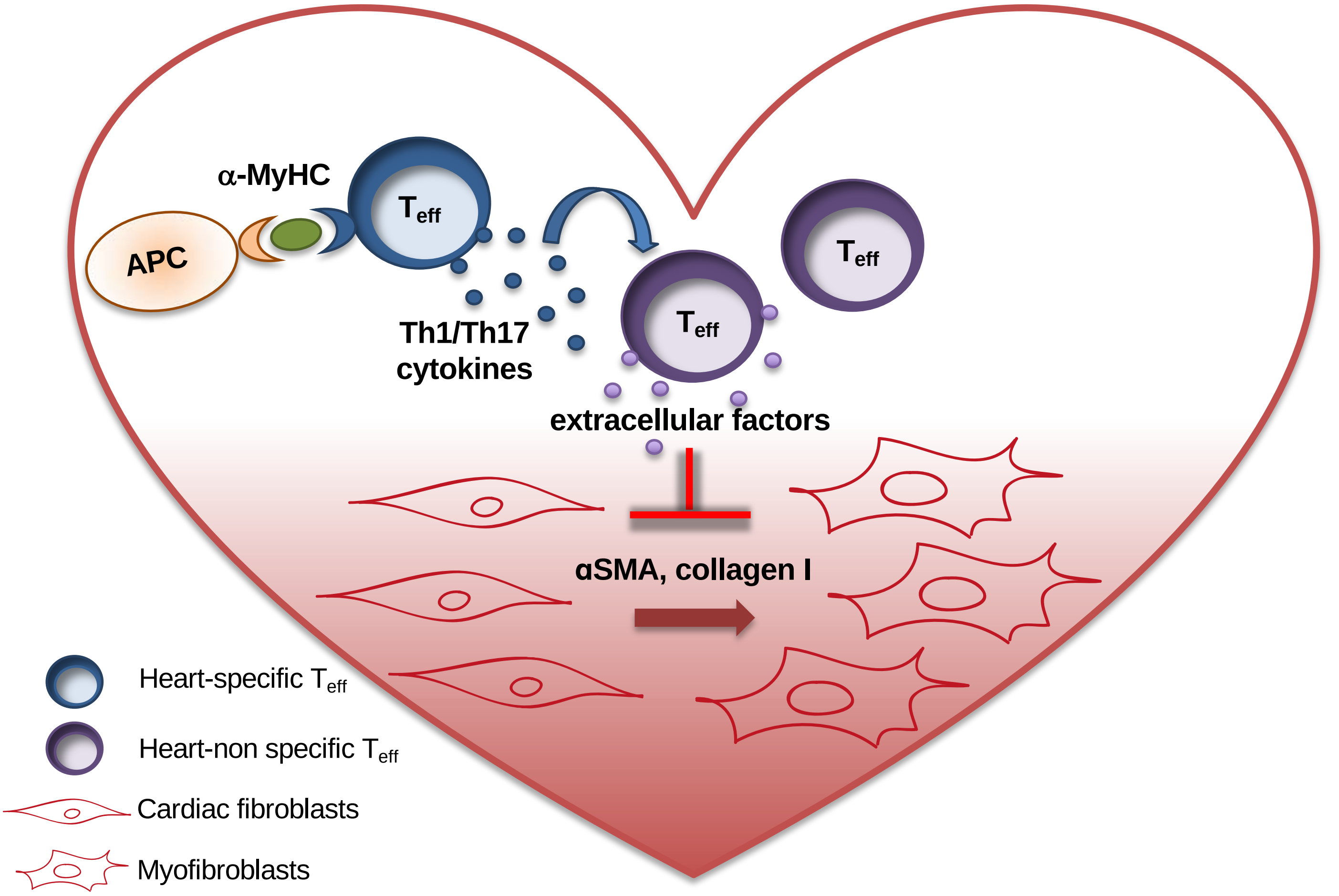

Supplementary Figure 11. Proposed antifibrotic mechanisms mediated by heart non-specific $\mathbf{T_{eff}}$. Heart-specific **α**-MyHC-reactive T_{eff} are activated by their cognate antigen through the TCR, produce Th1/Th17 cytokines and induce myocarditis. In response to inflammation, heart non-specific T_{eff} migrate into inflamed myocardium and secrete extracellular factors. Over time heart non-specific T_{eff} partially replace **α**-MyHC-reactive T_{eff} in myocarditis and Th1/Th17 cytokines are replaced by the secretome of heart non-specific T_{eff}. Accordingly, this switch in cytokine profile might prevent fibroblast-to-myofibroblast transformation and thus suppress fibrotic changes in the postinflammatory heart. APC – antigen presenting cell; T_{eff} – effector $CD4^+$ T cell; αSMA – alpha smooth muscle actin.

Supplementary Figure 11
