# Supplementary material for: Heart non-specific effector CD4+ T cells protect from postinflammatory fibrosis and cardiac dysfunction in experimental autoimmune myocarditis
Source: Basic Res Cardiol. 2019 Dec 20;115(1):6. doi: 10.1007/s00395-019-0766-6 (PMC6925074; doi:10.1007/s00395-019-0766-6)
Supplement: Supplementary file 2 — Supplementary material 2 (DOCX 59 kb) [file 395_2019_766_MOESM2_ESM.docx]

**SUPPLEMENTAL MATERIAL**

**Supplemental methods**

**Mice** All wildtype (n=209) and transgenic mice were maintained for at least 10 generations on a BALB/c background. TCR-M*-tg* crossed with Thy1.1-*tg* (CD90.1^+^, n=27)^1^, Ptprc^a^ (CD45.1-*tg*)^2^ (n=33) and Tg(DO11.10)10Dlo (DO11.10-*tg*)^3^ (n=91) mice were described previously. Mice are kept under standard laboratory conditions:12/12-h light/dark cycle, room temperature 20-22 ºC, humidity 45-55 % with access to food and water ad libitum. All animal experiments were performed in accordance with Swiss and Polish law and were approved by local authorities with permission numbers ZH194/12, ZH208/2105, ZH22/2106, SG16/07, SG17/07 (all Switzerland) and 313/2019 (Poland). All animal experiment followed the Guide for the Care and Use of Laboratory Animals, published by the US National Institutes of Health (NIH Publication, 8th Edition, 2011).

**EAM induction and adoptive T cell transfer** EAM was induced in 6-8 weeks old BALB/c or CD45.1-*tg* mice by subcutaneously injecting 200 µg of α-MyHC_614-634_ peptide (Ac-RSLKLMATLFSTYASADR-OH, Caslo) emulsified 1:1 with Complete Freund's Adjuvant (CFA, Difco) on days 0 and 7. In the respective experiments, EAM mice were injected intravenously with 3-5x10^6^ DO11.10^+^ T_eff_ , DO11.10^+^ T_n_ or TCR-M T_eff_ on days 17 and 20. For adoptive transfer, 6-8 weeks old BALB/c mice were sub-lethally irradiated (5.5 Gy) using a Gammatron (Co-60) and intravenously injected with CD45.1^+^ T_eff_, TCR-M T_eff_ and/or DO11.10^+^ T_eff_ (3-5x10^6^ of each cell type).

**Generation of T_eff_** Mouse T_eff_ were obtained from erythrocyte-lysed splenocytes/iLN cells, isolated from α-MyHC/CFA immunized CD45.1-*tg* mice at day 17 (CD45.1^+^ T_eff_), untreated TCR-M-*tg* (TCR-M T_eff_) or untreated DO11.10-*tg* (DO11.10^+^ T_eff_) mice. Splenocytes were stimulated with the respective antigen: CD45.1-*tg* cells with 5 µg/mL α-MyHC_614-634_, TCR-M-*tg* cells with 200 ng/mL α-MyHC_614-629_ and DO11.10-*tg* cells with 4 µg/mL OVA_323-339_ peptide (Anawa) in RPMI medium supplemented with 10% fetal calf serum (FCS, Gibco), 100.000 U/mL penicillin/streptomycin, 50 μM β-mercaptoethanol, 110 mg/L sodium pyruvate and non-essential amino acids (all Gibco) at 37ºC and 5% CO_2_ for 72 hours. CD4^+^ T cells purified by MACSorting using mouse anti-CD4 magnetic beads (Miltenyi) were used as T_eff_ for adoptive transfer experiments. For *in vitro* use, T_eff_ were additionally purified by FACSorting of CD4^+^CD44^hi^CD62L_low_ cells. DO11.10^+^ T_n_ were isolated from spleens of DO11.10-*tg* mice as described and directly (without *ex vivo* stimulation) purified using CD4 positive MACSorting and FACSorting for CD4^+^CD44_low_CD62L^hi^ cells using FACS Aria III.

Human CD4^+^ T cell subsets were sorted from peripheral blood mononuclear cells (PBMCs) derived from peripheral blood buffy coats of healthy donors (Blutspende Zurich, Switzerland), obtained with informed consent in accordance with the Declaration of Helsinki. PBMCs were isolated using Lympholyte (Cederlane) density gradient separation according to manufacturer’s instructions. The CD4^+^ T cell population was enriched by MACSorting using human anti-CD4 magnetic beads (Miltenyi). CD4^+^ T cell subpopulations were further sorted using FACS Aria III 4L cell sorter based on respective surface marker phenotypes: CD45RA^+^CD45RO^-^CCR7^+^CD27^+^ (T_n_) and CD45RA^-^CD45RO^+^CCR7^-^CD27^-^ (T_eff_).

**CD4^+^ T cell proliferation** CD4^+^ T cells were isolated from mouse erythrocyte-lysed splenocytes/LNs or human PBMCs by MACS or sorted from hearts digested with Liberase (Roche). CD4^+^ T cell subpopulations were further isolated using FACS Aria III 4L cell sorter. Typically, 5-20 000 T_eff_ were obtained from one heart with macroscopically visible myocarditis (only these hearts were used for CD4^+^ T cell recovery). Typically, cells from 2-3 hearts were pooled for one sample. Sorted cells were labeled with 2,5 μM CFSE or 5 μM CellTrace Violet Cell Proliferation Kit (both Life Technologies) according to manufacturer’s recommendations and either injected into recipient mice or cultured for 3-7 days in 96-well round-bottom plates (20 or 50x10^3^ cells/well) in RPMI medium supplemented with 10% FCS, penicillin/streptomycin, β-mercaptoethanol, sodium pyruvate, and non-essential amino acids at 37ºC and 5% CO_2_. For antigen-dependent activation, the respective CD4^+^ T cells were stimulated with 5 µg/mL α-MyHC_614-634_, 10-100 ng/mL α-MyHC_614-629_, or 4 µg/mL OVA_323-339_ peptide in the presence of APCs obtained from spleens of Rag2^-/-^ mice. 1:2 ratio of CD4^+^ T cells:APCs were used for *ex vivo* stimulation. Alternatively, cells were stimulated with anti-CD3/CD28 microbeads (Invitrogen) according to manufacturer’s recommendations. For antigen-independent responses, CD4^+^ T cell subpopulations were stimulated with IL-2, IL-7, IL-15, IL-21 (20 ng/mL each, all Peprotech). Cell proliferation was analyzed with LSRII Fortessa analyzer and FlowJo software (Tree Star) using the Proliferation platform.

**Cardiac fibroblasts isolation and culture** Mouse neonatal cardiac fibroblasts were obtained from heart ventricles of 1-3 days old neonatal BALB/c mice digested with Trypsin and Collagenase type II after pre-plating as previously described^4^. Human cardiac fibroblasts were generated by outgrowth from endomyocardial biopsies and characterized as fibroblasts as described previously^5,6^. Biopsies were obtained from left ventricles of heart failure patients suffering from reduced ejection fraction (EF <40%), under approval of local authorities and with informed consent. Primary cardiac fibroblasts were cultured in Iscove’s modified Dulbecco’s medium (Sigma) supplemented with 10% human serum, 10% FCS, 2 mM L-Glutamine, 10.000 U /mL penicillin/streptomycin (all Gibco). All experiments were performed with primary cells between second and fourth passage.

**Co-culture of** **CD4^+^ T cell with cardiac fibroblast** CD4^+^ T cell subpopulations were sorted from mouse or human MACS enriched CD4^+^ cells with FACS Aria III 4L cell sorter and cultured on confluent mouse or human cardiac fibroblasts, respectively. Co-culture was maintained in 24-well plates (10^5^ CD4^+^ T cells/well) with or without cell inserts (0.4 μm pore size polyester membrane, Corning) for gene expression analysis, intracellular protein staining and contraction assay; or in Nunc Lab Tek 8 chamber slides (5x10^4^ CD4^+^ T cells/well; Thermo Scientific) and 96 well black/clear flat bottom plates (5x10^4^ CD4^+^ T cells/well; Corning) for immunofluorescence imaging. Cells were kept in 1% FCS Iscove’s modified Dulbecco’s medium for 1-7 days. CD4^+^ T cells were stimulated with 20 ng/mL recombinant IL-2, IL-7, IL-15, IL-21 (20 ng/mL each). Prior fibroblast analyses, CD4^+^ T cells were removed from co-cultures by washing with PBS.

**Flow cytometry and cell sorting** Single cell suspensions were prepared either from digested mouse hearts treated for 45 min with 0.2 mg/mL Liberase (Roche), erythrocyte-lysed splenocytes or LNs using 70µm and 40µm cell strainers. Murine and human blood mononuclear cells were isolated using Lympholyte (Cedarlane). Freshly isolated or cultured cells were subjected to CD4 positive MACSorting and/or stained with the appropriate combination of fluorochrome-conjugated mouse antibodies: anti-CD45 FITC (clone 30-F11), anti-CD4 APC (RM4-5), anti-DO11.10-TCR FITC (KJ1-26), anti-CD45.1 PE (A20), anti-CD45.2 eFluor450 (104, all BD Bioscience), anti-CD44 APC-eFluor780 (IM7), CD90.1 APC-eFluor780 (HISS1, all eBioscience), anti-CD62L PE (MEL-14), anti-CD3 PerCP/Cy5.5 (17A2, all BioLegend). Human CD4^+^ T cells were stained with the appropriate combination of fluorochrome-conjugated human antibodies: anti-CD4 APC (EDU-2), anti-CD3 FITC (HIT3b, all ImmunoTools), anti-CD45RA eFluor450 (clone HI100), anti-CD45RO PerCP eFluor770 (UCHL1), anti-CCR7 PE (3D12), anti-CD27 APC eFluor770 (O323, all eBioscience). Prior to flow cytometry analysis, human CD4^+^ T cells were fixed with IC Fixation buffer (eBiosicence) diluted in 1:4 in PBS. Cell viability was quantified with propidium iodide for non-fixed cells or with Viability dye eFluor506 for fixed cells (both eBioscience). Apoptosis was measured using the Annexin-V detection kit (BioLegend). Cells were analyzed with the LSRII Fortessa analyzer (BD Bioscience). In the respective experiments, CD4^+^ T cell fractions were sorted with FACSAria III (BD Bioscience, obtained purity >98%).

**Intracellular flow cytometry** For intracellular protein staining, human cardiac fibroblasts were fixed with 4% paraformaldehyde, permeabilized with 0.1% Triton (both Sigma) and stained with anti-mouse/human αSMA (Sigma) and biotin conjugated anti-human collagen I (Acris) antibodies, followed by staining with secondary Alexa Flour-488 goat anti-mouse antibody (Invitrogen) and BD Horizon V450 Streptavidin (BD Bioscences), respectively. Cells were analyzed with LSRII Fortessa analyzer.

**Immunocytochemistry** Human and mouse cardiac fibroblasts were fixed with 4% paraformaldehyde, followed by permeabilization with 0,1% Triton. Cells were stained with anti-mouse/human αSMA primary antibody, followed by secondary goat anti-mouse Alexa Flour-488 and Phalloidin (Sigma). Nuclei were stained with DAPI. Immunofluorescence was analyzed using a Leica DM IRE2 microscope equipped with the Nomarski Interference Contrast and Operetta high-content screening platform **(**Perkin-Elmer) as described previously^7^.Quantification of αSMA in human cardiac fibroblasts was performed using ImageJ software by counting at least 200 F-actin-positive cells (stained with phalloidin) per sample. Quantification of αSMA fibers in mouse cardiac fibroblasts was performed using the Harmony software (Perkin-Elmer) by measuring the mean fluorescence intensity of 5 images per sample.

**Transthoracic echocardiography and Doppler imaging** Transthoracic echocardiography was performed using a Vevo 2100 system equipped with 30-MHz transducer (VisualSonics). Anesthesia was induced by 5% isoflurane and confirmed by the absence of the withdrawal reflex of one of the hind paws. During echocardiogram acquisition isofluorane was reduced to 1.5-2%. Each animal was placed in a supine position on a prewarmed platform. The limbs were taped over the metal ECG leads to enable continuous monitoring of the heart rate and respiration. Then, the prewarmed echo transmission gel was applied to the hairless chest. The heart was imaged in the bidimensional (2-D) mode, in the parasternal long-axis (PSLAX), short-axis (SAX) and apical 4-chamber views. For analysis of left ventricular end-diastolic volume (LV vol, d) and left ventricular end-systolic volume (LV vol, s) the endocardium of the left ventricle was traced at both diastole and systole. An integrated software tool (LV-Trace) was used for single-plane PSLAX analysis (see Suppl. Fig. 7). Collected parameters include: heart rate (HR), ejection fraction (EF), stroke volume (SV), cardiac output (CO), left ventricular end-diastolic diameter (LVID, d), left ventricular end-systolic diameter (LVID, s), fractional shortening (FS), peak Doppler blood inflow velocity across the mitral valve during early diastole (E), peak Doppler blood inflow velocity across the mitral valve during late diastole (A).

**Histopathology and Immunohistochemistry** Mouse heart tissues were fixed in 4% formalin and embedded in paraffin. Conventional Hematoxylin/Eosin and Masson’s trichrome staining were used to assess cardiac inflammation and fibrosis, respectively, as previously described^3,8^. Antigen retrieval was performed using ER2 buffer (Leica) and sections were stained with anti-mouse CD45.1, anti-mouse CD90.1 (both eBioscience), anti-mouse CD45 (BD Bioscience), anti-mouse CD3 (Neomarkers), anti-mouse αSMA (clone E184, Abcam), anti-mouse periostin (Abcam) or anti-mouse vimentin (Abcam) antibodies, followed by anti-mouse-HRP or anti-rabbit-HRP and chromogenic DAB staining. Counterstaining was performed using Hematoxylin. Immunopositive cells and areas were quantified using Olympus BX51 microscope and cellSens (Olympus) or Fiji software with custom-made plug-ins.

Formalin fixed, paraffin embedded human heart tissue from 10 acute lymphocytic myocarditis patients was obtained from a Biobank of the Institute for Pathology and Neuropathology, University Hospital Tubingen in accordance with local ethical regulations. Heat-induced antigen retrieval was performed using Tris-EDTA buffer, pH=9. Unspecific binding was prevented by blocking with 5% BSA (Sigma), 5% goat serum (Abcam), 0.025% Triton-X (Sigma) in PBS. Sections were stained using rabbit anti-human CD4 (EPR6855, Abcam), mouse anti-human CD45RO (UCHL-1, eBioscience) and mouse anti-human CD45RA (HI100, eBioscience), followed by secondary anti-mouse Alexa Fluor-546 and anti-rabbit Alexa Fluor-647 (both Invitrogen) staining. Nuclei were stained with DAPI. Immunofluorescence was analyzed using Zeiss AxioObserver Z1 widefield microscope and processed by ImageJ.

**Hydroxyproline assay** Cardiac tissue was digested in distilled water (100 µL per 10mg of tissue) using Precellys Soft tissue homogenizing CK14 beads and tissue homogenizer Precellys 24 (all Precellys) for 2x6000 rpm, 20 sec each. Homogenate was spun down for 2 min at maximum speed and 100 µL of supernatant was transferred to 2 mL Teflon capped pressure tight vials (Supelco, Sigma). After addition of 100 µL 12N hydrochloric acid (Sigma), samples were hydrolyzed for 3 h at 120 ºC in an oven. Hydrolyzed samples were spin down at 1000xg for 5 min. 10 µL of supernatant was transferred to a 96 well plate and evaporated to dryness at 60 ºC in an oven for 30-45 min. Samples were further processed for hydroxyproline assay according to manufacturer’s instructions (Hydroxyproline Assay Kit, Cell Biolabs). Absorbance was measured at 540-560 nm and concentration was determined using the hydroxyproline standard curve.

**Contraction assay** Fibroblast contraction was measured using the Cell Contraction Assay (Cell Biolabs), as previously described^8^. Prior plating into the collagen matrix, human cardiac fibroblasts were co-cultured with CD4^+^ T cell subpopulations in the presence of γc-cytokines for 7 days in 1% FCS Iscove’s modified Dulbecco’s medium. Cell contraction was measured at day 5 following the manufacturer’s protocols using the Amersham Imager 600 (GE Healthcare Life Sciences).

**ELISA** Cytokine levels in cell culture supernatants were measured according to the manufacturer’s protocol using mouse IFN-γ, IL-17A, IL-2, IL-4, GM-CSF, MCP1, and MIP1α ELISA kits (all eBioscience).

**Quantitative RT-PCR** Prior RNA isolation, 25-30 mg of cardiac tissue was lysed in 400 µL of lysis buffer and homogenized using Precellys Soft tissue homogenizing CK14 beads and tissue homogenizer Precellys 24 (all Precellys) for 2x6000 rpm, 20 sec each. Homogenate was spun down for 2 min at maximum speed and the supernatant was processed according to the manufacturer’s instructions (High Pure RNA Tissue isolation kit, Roche). Total RNA from cells was isolated using the High Pure RNA isolation kit (Roche) following manufacturer’s instructions. RNA concentration was measured using NanoDrop (TermoFischer) and 150-200 ng of RNA was used for cDNA synthesis using RevertAid Reverse Transcriptase and Oligo(dT)_18_ (both Thermo Scientific). cDNA was amplified with the QuantStudio 6 Flex Real-Time PCR System (Applied Biosystems) using Power SYBR Green PCR Master Mix (Applied Biosystems) and oligonucleotides for the genes of interest: *gapdh* F 5’-CTGCACCACCAACTGCTTAGC-3’, R 5’-GGCATGGACTGTGGTCATGAG-3’; *acta2* F 5’-CGCTGTCAGGAACCCTGAGA-3’, R 5’-ATCCCAGCCTCCGTTATCCT-3’; *col1a1* F 5’-GATGACGTGCAATGCAATGAA-3’, R 5’-CCCTCGACTCCTACATCTTCTGA-3’; *fn1* F 5’-TACCAAGGTCAATCCACACCCC-3’, R 5’-GAGATGGCAAAAGAAAGCAGAGG-3’; *vim* F 5'-GCCGAGGAATGGTACAAGTCCAAG-3', R 5'-GGG CAT CCA CTT CAC AGG TGA G-3'; *GAPDH* F 5’-GGGAAGCTTGTCATCAATGGA-3’, R 5’-TCTCGCTCCTGGAAGATGGT-3’; *ACTA2* F 5'-ATGCCATGTTCTATCGGGTACTT -3', R 5'- GACAATGGCTCTGGGCTCTGTAA-3'; COL1A1 F 5'-TTTTGTATTCAATCACTGTCTTGCC-3', R 5'-CAGCCGCTTCACCTACAGC-3'; FN1 F 5'-GGAGAATTCAAGTGTGACCCTCATG -3', R 5'-TGCCACTGTTCTCCTACGTGG -3'; *VIM* F 5'-GCT TCA GAG AGA GGA AGC CG-3', R 5'-AAG GTC AAG ACG TGC CAG AG-3' (Microsynth). Transcript levels of *gapdh* (for mouse) or *GAPDH* (human) were used as endogenous reference and where relevant, relative gene expression was analyzed using the 2^-ΔΔCt^ method.

**The whole genome transcriptomics** RNA was isolated from sorted T_eff_ subsets (30-40 000 cells) using the High Pure RNA RNA isolation kit (Roche). RNA quality was determined using the 4200 TapeStation (Agilent) and transcriptomics profiling was performed using Clontech SMART-Seq Ultra Low Input mammalian total RNA library kit (Takara) and HiSeq4000 (Illumina) by the Functional Genomics Center Zurich. The data are available at the European Nucleotide Archive (https://www.ebi.ac.uk/ena) with the accession number PRJEB28775. The data were analyzed using the FGCZ Heatmap online platform: http://fgcz-shiny.uzh.ch/fgcz_heatmap_app/

**Statistics** Where relevant, the data were analyzed by unpaired, two-tailed Student’s *t*-test, one-way ANOVA followed by the Dunnett *post-hoc* test for normally distributed data or Mann-Whitney test (for nonparametric data). Differences were considered statistically significant for p<0.05. All analyses were performed with GraphPad Prism 6 software and values are expressed as mean with SEM.

**Supplementary Table 1.** Clinical characteristics of myocarditis patients for assessment of heart-infiltrating CD4^+^ T cell phenotype in endomyocardial biopsies.

| Patient | Clinical  Diagnosis  Myocarditis | Gender | Birth | Age | Lymphocytic  Myocarditis | Eosinophilic/  Giant cell  Myocarditis | Viral  Genome | DCM^*^ | EF^†^ |
| --- | --- | --- | --- | --- | --- | --- | --- | --- | --- |
| 1 | acute | female | 1989 | 27 | pos | neg | neg | neg | 35% |
| 2 | acute | male | 1950 | 66 | pos | neg | neg | neg | 15% |
| 3 | acute | male | 1976 | 40 | pos | neg | neg | neg | 35% |
| 4 | acute | female | 1961 | 55 | pos | neg | neg | neg | 40% |
| 5 | acute | female | 1968 | 48 | pos | neg | neg | neg | 20% |
| 6 | acute | male | 1981 | 35 | pos | neg | neg | neg | 55% |
| 7 | acute | male | 1993 | 23 | pos | neg | neg | neg | 40% |
| 8 | acute | female | 1963 | 49 | pos | neg | neg | neg | 46% |
| 9 | acute | male | 1991 | 21 | pos | neg | neg | neg | 15% |
| 10 | acute | male | 1976 | 36 | pos | neg | neg | neg | 8% |

^*^DCM - dilated cardiomyopathy; ^†^EF - ejection fraction

**Supplementary Table 2.** Echocardiography analyses of cardiac function of BALB/c mice before induction of EAM (d0) and 40 days after EAM induction. The mice were additionally treated either with PBS or DO11.10^+^ T_eff_ cells at days 17 and 20 of EAM. Abbreviations: bpm: beat per minute; LV vol, d: left ventricular end diastolic volume; LV vol, s: left ventricular end systolic volume; EF: ejection fraction; SV: stroke volume; CO: cardiac output; LVID, d: left ventricular end diastolic internal diameter; LVID, s: left ventricular end systolic internal diameter; FS: fractional shortening; MV E/A: peak Doppler blood inflow velocity across the mitral valve during early diastole (E) to peak Doppler blood inflow velocity across the mitral valve during late diastole (A) ratio.

| Parameter | Day 0 | | Day 40 | |
| --- | --- | --- | --- | --- |
|  | PBS | DO11.10^+^ T_eff_ | PBS | DO11.10^+^ T_eff_ |
|  | Mean ± SD | Mean ± SD | Mean ± SD | Mean ± SD |
| heart rate [bpm] | 357.9 ± 23.7 | 347.3 ± 38.0 | 325.8 ± 65.6 | 345.8 ± 24.1 |
| LV vol, d [μl] | 60.2 ± 5.8 | 61.8 ± 11.5 | 71.7 ± 15.5 | 65.4 ± 11.4 |
| LV vol, s [μl] | 25.3 ± 2.4 | 25.8 ± 6.7 | 43.3 ± 12.4 | 30.9 ± 9.9 |
| EF [%] | 57.7 ± 4.6 | 58.6 ± 4.5 | 40.3 ± 9.0 | 53.2 ± 8.0 |
| SV [μl] | 34.9 ± 5.7 | 36.1 ± 5.9 | 43.3 ± 12.4 | 34.5 ± 6.0 |
| CO [ml/min] | 12.6 ± 2.6 | 12.6 ± 2.7 | 9.4 ± 3.2 | 12.0 ± 2.4 |
| LVID, d [mm] | 4.0 ± 0.2 | 4.0 ± 0.3 | 4.1 ± 0.4 | 3.9 ± 0.2 |
| LVID, s [mm] | 2.8 ± 0.2 | 2.8 ± 0.3 | 3.3 ± 0.6 | 2.9 ± 0.3 |
| FS [%] | 29.3 ± 3.4 | 27.8 ± 3.2 | 18.4 ± 7.1 | 25.5± 5.9 |
| MV E/A | 1.9 ± 0.4 | 2.1 ± 0.5 | 3.2 ± 1.9 | 2.5 ± 0.9 |

**Supplemental references**

1. Nindl V, Maier R, Ratering D, De Giuli R, Züst R, Thiel V, Scandella E, Di Padova F, Kopf M, Rudin M, Rülicke T and Ludewig B. Cooperation of Th1 and Th17 cells determines transition from autoimmune myocarditis to dilated cardiomyopathy. *Eur J Immunol*. 2012;42:2311-21.

2. Kania G, Blyszczuk P, Valaperti A, Dieterle T, Leimenstoll B, Dirnhofer S, Zulewski H and Eriksson U. Prominin-1+/CD133+ bone marrow-derived heart-resident cells suppress experimental autoimmune myocarditis. *Cardiovasc Res*. 2008;80:236-45.

3. Kania G, Siegert S, Behnke S, Prados-Rosales R, Casadevall A, Lüscher TF, Luther SA, Kopf M, Eriksson U and Blyszczuk P. Innate signaling promotes formation of regulatory nitric oxide-producing dendritic cells limiting T-cell expansion in experimental autoimmune myocarditis. *Circulation*. 2013;127:2285-94.

4. Leenders JJ, Wijnen WJ, Hiller M, van der Made I, Lentink V, van Leeuwen RE, Herias V, Pokharel S, Heymans S, de Windt LJ, Høydal MA, Pinto YM and Creemers EE. Regulation of cardiac gene expression by KLF15, a repressor of myocardin activity. *J Biol Chem*. 2010;285:27449-56.

5. Lindner D, Zietsch C, Becher PM, Schulze K, Schultheiss HP, Tschöpe C and Westermann D. Differential expression of matrix metalloproteases in human fibroblasts with different origins. *Biochem Res Int*. 2012;2012:875742.

6. Westermann D, Lindner D, Kasner M, Zietsch C, Savvatis K, Escher F, von Schlippenbach J, Skurk C, Steendijk P, Riad A, Poller W, Schultheiss HP and Tschöpe C. Cardiac inflammation contributes to changes in the extracellular matrix in patients with heart failure and normal ejection fraction. *Circ Heart Fail*. 2011;4:44-52.

7. Paw M, Borek I, Wnuk D, Ryszawy D, Piwowarczyk K, Kmiotek K, Wójcik-Pszczoła KA, Pierzchalska M, Madeja Z, Sanak M, Błyszczuk P, Michalik M and Czyż J. Connexin43 Controls the Myofibroblastic Differentiation of Bronchial Fibroblasts from Patients with Asthma. *Am J Respir Cell Mol Biol*. 2017;57:100-110.

8. Blyszczuk P, Müller-Edenborn B, Valenta T, Osto E, Stellato M, Behnke S, Glatz K, Basler K, Lüscher TF, Distler O, Eriksson U and Kania G. Transforming growth factor-β-dependent Wnt secretion controls myofibroblast formation and myocardial fibrosis progression in experimental autoimmune myocarditis. *Eur Heart J*. 2016.
